# Aberrant transcriptional regulations in cancers: genome, transcriptome and epigenome analysis of lung adenocarcinoma cell lines

**DOI:** 10.1093/nar/gku885

**Published:** 2014-11-06

**Authors:** Ayako Suzuki, Hideki Makinoshima, Hiroyuki Wakaguri, Hiroyasu Esumi, Sumio Sugano, Takashi Kohno, Katsuya Tsuchihara, Yutaka Suzuki

**Affiliations:** 1 Department of Medical Genome Sciences, Graduate School of Frontier Sciences, The University of Tokyo, Chiba, Japan; 2 Division of TR, The Exploratory Oncology Research and Clinical Trial Center, National Cancer Center, Chiba, Japan; 3 Department of Computational Biology, Graduate School of Frontier Sciences, The University of Tokyo, Chiba, Japan; 4 Division of Genome Biology, National Cancer Center Research Institute, Tokyo, Japan; 5 Division of TR, The Exploratory Oncology Research and Clinical Trial Center, National Cancer Center, Tokyo, Japan

## Abstract

Here we conducted an integrative multi-omics analysis to understand how cancers harbor various types of aberrations at the genomic, epigenomic and transcriptional levels. In order to elucidate biological relevance of the aberrations and their mutual relations, we performed whole-genome sequencing, RNA-Seq, bisulfite sequencing and ChIP-Seq of 26 lung adenocarcinoma cell lines. The collected multi-omics data allowed us to associate an average of 536 coding mutations and 13,573 mutations in promoter or enhancer regions with aberrant transcriptional regulations. We detected the 385 splice site mutations and 552 chromosomal rearrangements, representative cases of which were validated to cause aberrant transcripts. Averages of 61, 217, 3687 and 3112 mutations are located in the regulatory regions which showed differential DNA methylation, H3K4me3, H3K4me1 and H3K27ac marks, respectively. We detected distinct patterns of aberrations in transcriptional regulations depending on genes. We found that the irregular histone marks were characteristic to EGFR and CDKN1A, while a large genomic deletion and hyper-DNA methylation were most frequent for CDKN2A. We also used the multi-omics data to classify the cell lines regarding their hallmarks of carcinogenesis. Our datasets should provide a valuable foundation for biological interpretations of interlaced genomic and epigenomic aberrations.

## INTRODUCTION

Lung cancer is one of the most significant causes of death in the world. In particular, lung adenocarcinoma is the most commonly occurring lung cancer. Previous studies have identified several genes whose aberrations are responsible for carcinogenesis, such as TP53, CDKN2A, KRAS and EGFR (1–3). EGFR-activating mutations are more prevalent in female, never-smokers and Asians (4,5). These mutations have become a target for molecular targeting drugs, gefitinib and erlotinib (6). Also, gene fusions between the ALK, RET and ROS1 oncogenes and other partner genes, producing oncogenic fusion transcripts, have been identified as causative ‘driver’ aberrations. These fusions are involved in carcinogenesis in a fraction (1–5%) of lung adenocarcinoma (7–11). The fact that many of such fusion genes have been discovered by transcriptome analysis has re-enforced the importance in investigating the lung cancers also from the viewpoint of transcriptome.

Recently, a global view of genome aberrations in lung and other cancers are being obtained by next-generation sequencing analysis of cancer tissues by The Cancer Genome Atlas (TCGA) (12–14) and The International Cancer Genome Consortium (ICGC) (15). These intensive studies have demonstrated that the mutation patterns and disrupted pathways are highly diverse between cancer types and patients. For lung adenocarcinoma, large datasets collected from several groups, including ours (2–3,16), have revealed that the number and patterns of mutations were some of the most complex signatures among all cancer types.

In spite of the rapid accumulation of cancer genome data, the current view of cancer biology is still far from perfect. Recent studies have revealed that gene expression profiles of cancer cells, which underlie phenotypic appearances of cancer cells, are consequences not only of genome aberrations but also of aberrations in DNA methylation and chromatin statuses. Indeed, recent analyses have indicated that aberrations in the epigenome and transcriptome regulators play pivotal roles in carcinogenesis. The mutations in the genes that have regulatory roles in gene expression have been reported in lung and other cancers, such as chromatin remodeling factors (e.g. ARID1A/BAF250A and SMARCA4/BRG1) and splicing factors (e.g. U2AF1 and RBM10) (2,14,17). However, despite the claimed importance, it remains elusive as to which genomic and epigenomic aberrations have biological relevance among transcriptomic aberrations and how they collectively contribute to cancer phenotypes. This is mainly due to a general lack of transcriptome and epigenomic information that is directly associated with genomic aberrations. Technical difficulties are frequently inevitable when clinical tumor samples are used for transcriptomic and, particularly, epigenomic analyses. Unlike normal tissues, which are being used for several projects, such as the NIH Roadmap Epigenomics Mapping Consortium (18), the amount of available clinical cancer tissue is small, mixed with normal tissue, and more importantly, not suitable for ChIP-Seq analyses. On the other hand, the utility of cultured cancer cell lines has been established in omics analyses. In fact, the Encyclopedia of DNA Elements (ENCODE) consortium project (19,20) analyzed several representative cultured cells and generated a comprehensive view of human genome, epigenome and transcriptome. The information has greatly improved our system-level understandings of how various regulatory factors are orchestrated to determine downstream gene expression levels and demonstrated their variations between different cell types.

In the present study, 26 human lung cancer cell lines were subjected to multi-omics analyses to generate a reference for omics information. We expected this informational resource should be useful to investigate clinical lung cancers, also providing a tool for future biological assays. Indeed, we demonstrated that integrative analysis of the multilayer-omics resource has revealed various irregular patterns of regulatory factors. Unexpectedly, we found that the aberrant expression was associated with various causative events, which are characteristically gene-dependent. Here, we describe the generation and utilization of our unique multi-omics catalog of lung adenocarcinoma cell lines.

## MATERIALS AND METHODS

### Data access

All raw sequence data were deposited in the DNA Data Bank of Japan (DDBJ) with the accession number, DRA001859 and DRA001858 (whole-genome sequencing), DRA001846 (RNA-Seq), DRA001841 (bisulfite sequencing), DRA001860 (ChIP-Seq) and DRA002311 (ChIP-Seq and RNA-Seq of small airway epithelial cells (SAEC)). All datasets in this paper are also provided in the web database (URL: http://dbtss.hgc.jp/).

### Cell lines

Twenty-six lung adenocarcinoma cell lines were described in Supplementary Table S1. Cells were cultured in the RPMI medium (RPMI 1640, Nissui), Dulbecco's Modified Eagle's medium (Nissui) or Eagle's minimal essential medium (Nissui) supplemented with 10% FBS, MEM Non-essential Amino acid solution (SIGMA) and antibiotics (Antibiotic-Antimycotic, GIBCO) in an incubator maintained at 37°C and 5% CO_2_. Four cancer cell lines (LC2/ad, PC-3, H1648 and H2347) were cultured using collagen-coated dishes (collagen Type I-coated, IWAKI). Normal human SAEC (CC-2547, Takara) were also cultured in the SAGM BulletKit (CC-3118, Takara) using collagen-coated dishes.

### Whole-genome sequencing and RNA-Seq

Cultured cells were harvested and washed with phosphate buffered saline (PBS). DNA purification was performed using the DNeasy Kit (QIAGEN). Using the isolated DNA, we prepared libraries and performed whole-genome sequencing using the HiSeq platform (Illumina) according to the manufacturer's protocol. RNA was extracted from the harvested cells using the RNeasy Maxi Kit (QIAGEN). We prepared RNA-Seq libraries and performed sequencing using the HiSeq platform according to the manufacturer's protocol.

### Target-captured bisulfite sequencing

Using 3 μg of isolated DNA, we prepared the bisulfite-converted DNA libraries using the SureSelect Methyl-Seq Target Enrichment System (Agilent Technologies) and EZ-DNA Methylation-Gold Kit (Zymo Research) according to each manufacturer's protocol. The DNA was sequenced using the HiSeq platform.

### ChIP-Seq

We performed ChIP-Seq (21,22) for RNA Polymerase II and seven histone modifications using the following antibodies; anti-RNA Polymerase II (ab817, Abcam), anti-H3K4me1 (ab8895, Abcam), anti-H3K4me3 (ab1012, Abcam), anti-H3K9me3 (ab8898, Abcam), anti-H3K27me3 (07–449, Millipore; ab6002, Abcam), anti-H3K36me3 (ab9050, Abcam), anti-H3K9/14ac (06–599, Millipore) and anti-H3K27ac (ab4729, Abcam). Each antibody (10 μg or 20 μg of anti-H3K27me3) was added to the magnetic beads (Dynabeads Protein G/A, Invitrogen) with the blocking buffer (0.5% bovine serum albumin in PBS solution) and rotated for more than 4 h at 4°C. Cultured cancer cells (1 × 10^7^–1 × 10^8^ cells) were crosslinked in 1% (0.5% for PC-7) formaldehyde solution and incubated for 10 min at room temperature. To stop the fixation, 125 mM glycine was added to the dishes. The cells were incubated for 5 min at room temperature, washed using cold PBS and harvested using a scraper. Lysis buffer 1 (50 mM HEPES-KOH pH 7.5, 140 mM NaCl, 1 mM EDTA pH 8.0, 10% glycerol, 0.5% Nonidet P-40 and 0.25% Triton X-100), lysis buffer 2 (200 mM NaCl, 1 mM EDTA pH 8.0, 0.5 mM EGTA pH 8.0 and 10 mM Tris-HCl pH 8.0) and lysis buffer 3 (100 mM NaCl, 1 mM EDTA pH 8.0, 0.5 mM EGTA pH 8.0, 10 mM Tris-HCl pH 8.0, 0.1% sodium deoxycholate and 1% N-lauroylsarcosine) were prepared with protease inhibitor (Roche). The harvested cells were dissolved using cold lysis buffer 1 and incubated for 10 min on ice. The cells were centrifuged at 1500 rpm for 5 min and the pellet was redissolved using cold lysis buffer 2. The cells were incubated for 10 min on ice and centrifuged at 1500 rpm for 5 min. The collected pellet was lysed using cold lysis buffer 3 and cracked with 16 cycles (10 cycles for PC-7) of 30 s of sonication on ice. Triton X-100 (10%, 100 μl) was added to the sonicated samples. The cells were centrifuged at 14,000 rpm for 10 min and 50 μl of the supernatant was moved to a different 1.5 ml tube (whole-cell extract (WCE) sample). The magnetic beads with each antibody were washed using blocking buffer and added to the supernatant (ChIP sample). The sample was rotated at 4°C overnight for the immunoprecipitation. The sample was washed eight times using wash buffer (50 mM HEPES-KOH pH 7.5, 500 mM LiCl, 1 mM EDTA pH 8.0, 1% Nonidet P-40, 0.7% sodium deoxycholate) and once using TE buffer (50 mM Tris-HCl pH 8.0 and 10 mM EDTA pH 8.0) with 50 mM of NaCl. The sample was eluted in 200 μl of elution buffer (50 mM Tris-HCl pH 8.0, 10 mM EDTA pH 8.0 and 1% sodium dodecyl sulfate) and incubated for 15 min at 65°C. The supernatant was moved to a new 1.5 ml tube. Elution buffer (150 μl) was added to the WCE sample and then both ChIP and WCE samples were incubated for more than 6 h at 65°C to de-crosslink. TE buffer (200 μl) and 8 μl of 10 mg/ml RNase A (Novagen) were added to the samples and the samples were incubated for 2 h at 37°C. Proteinase K (20 mg/ml, 4 μl) (Takara) and 5 mM CaCl_2_ were added to the samples and they were incubated for 30 min at 55°C. The DNA samples were purified by phenol chloroform extraction and ethanol precipitation and finally eluted in 35 μl of water. Using DNA samples from the ChIP and WCE samples, we prepared ChIP-Seq libraries and performed sequencing using the HiSeq platform according to the manufacturer's protocol.

### Identification of single nucleotide variants and short indels

As shown in Supplementary Figure S1, whole-genome sequences were mapped to the human reference genome (UCSC hg19) by the Burrows-Wheeler Aligner (BWA) (23) after removing sequences with quality control (QC) failure and adapters. Using SAMtools (24), PCR duplicates were removed. The single nucleotide variants (SNVs) and insertion/deletions (indels) were detected by the Genome Analysis Toolkit (GATK) Unified Genotyper and Somatic Indel Detector (25,26). Using our Perl scripts, the SNVs were screened under the following condition: 4× or more variant sequences at the position of the SNVs. The indels were extracted under two parameters: (i) 4× or more variant sequences at the position of the indels and (ii) the variants detected from both the forward and reverse-strand sequences. The NCBI dbSNP build 137, the NHLBI Exome Sequencing Project (Exome Variant Server, 8 October 2013 accessed, URL: http://evs.gs.washington.edu/EVS/; allele frequency > 0.1%), the 1000 Genomes Project (allele frequency > 0.1%) and the in-house Japanese data were used to discriminate the known single nucleotide polymorphisms (SNPs) and to extract somatic SNVs and indels (27,28). Subsequently, SNVs and indels registered in COSMIC (release v59) were rescued as somatic mutation candidates (29,30).

### Copy number analyses

Genome-wide copy number information was obtained using Control-FREEC (31,32). We analyzed the genomes of the 26 cell lines as diploid and obtained the results for two window sizes, lower-resolution data (50 kb) and higher-resolution data (1.5 kb). The lower-resolution data were used to draw the figures of genome-wide copy number information and the higher-resolution data were used to detect gene-level copy number aberrations (CNAs). The regions with normalized copy numbers ≥ 4 or ≤ 1 were detected as copy number gains and losses, respectively.

### Detecting chromosome rearrangements

The obtained whole-genome sequences were mapped as single-end sequences by BWA. Mates spanning in different chromosomes or > 1 Mb of the same chromosome were used to search for ‘reference tags’ of each junction point supported by both directions. Next, ‘supporting tags’ were detected from all sequences, which were mapped on the ‘reference tags’. We extracted gene pairs with ≥ 2 ‘reference tags’ and ≥ 4 ‘supporting tags’ of the junction point. In addition, gene pairs uniquely occurring in each cell line were selected as rearrangement candidates.

### Generating gene expression profiles

The obtained RNA-Seq data were mapped to the human reference genome using ELAND (Illumina). For a total of 20,598 genes, parts per million mapped reads (PPM) and reads per kilo base per million mapped reads (RPKM) were calculated as an expression level of each gene using the Perl script. Expression abundances for the selected 52 genes were validated by qRT-PCR (Supplementary Figure S2). PCR primers were designed by Primer3Plus (33) (Supplementary Table S2A). Files for visualization of RNA-Seq on Integrative Genomics Viewer (IGV) (34,35) were created using TopHat2 (36).

### Detecting fusion transcripts

The obtained RNA-Seq data were mapped using TopHat2 with the following options; -r 50 -p 8 -no-coverage-search -mate-std-dev 80 -max-intron-length 100000 -fusion-min-dist 10000000 -fusion-anchor-length 13 -fusion-search -keep-fasta-order -bowtie1. Using the mapped RNA-Seq data, fusion transcript candidates were filtered by tophat-fusion-post (37) and extracted under the following conditions: ≥ 10 spanning reads and ≥ 2 spanning mate pairs. Several cases were validated by RT-PCR. PCR primers were designed using Primer3Plus and are shown in Supplementary Table S2B.

### Analyses of DNA methylation

The obtained sequences from bisulfite sequencing were mostly obtained from the antisense chain of the genome. We modified the sequences by the in-house Perl script (read1: C to T, read2: G to A). Using BWA, the modified sequences were mapped to the modified (G to A) human reference genome. According to the mapping results, pre-modified sequences were mapped on the genome and the following sites were counted: CG, CA, CT and CC with methylated-C and TG, TA, TT and TC with non-methylated-C. The C to T conversion rates were calculated using the C sites of non-CpG sites. All datasets satisfied 99% of the conversion rate. For the CpG sites, the ratios of CG to total depths in each site (≥ 5×) were calculated as methylation rates. The information on CpG islands used in this analysis was provided by the UCSC. DNA methylation rates of several cases were validated by direct Sanger sequencing (*n* = 3) and Sanger sequencing of TA cloning (pMD20-T, Takara) for individual clones (Supplementary Table S3 and Supplementary Figure S3). PCR primers are shown in Supplementary Table S2C.

For the genome-wide DNA methylation status, we calculated DNA methylation rates for each 50 kb of the human genome and performed hierarchical clustering for the 26 cell lines. For a total of 19,323 genes, DNA methylation rates of promoters, which were defined as up to 1.5 kb from the most upstream transcriptional start sites (TSSs), were also calculated. For the 26 cancer-related genes, we selected the representative TSS of each gene by manual inspection and also calculated the methylation rates of the promoters.

### Detecting patterns of histone modifications and RNA polymerase II binding profiles

All ChIP samples were validated by qPCR (Supplementary Table S4). ChIP-Seq data for each histone modification and RNA polymerase II binding were mapped to the human reference genome using ELAND (Illumina). Using MACS2 with default parameters (38,39), narrow peaks of each ChIP-Seq dataset were detected as the histone modification and Pol II binding patterns. Broad peaks were also detected by MACS2 for the repressive markers, H3K27me3 and H3K9me3. For the enhancer marks of H3K4me1 and H3K27ac, all narrow peaks of MACS2 from the 26 cell lines were gathered and classified depending on the positions and the representative enhancer regions were identified.

For a total of 20,598 genes, ChIP-Seq tag densities (fold of WCE) of the regions of ± 1.5 kb from most upstream TSSs and gene bodies were calculated as the intensities of each chromatin mark. To investigate the correlation among the chromatin statuses, we calculated the intensities of the gene and their proximal regions for each chromatin mark and Spearman's rank correlation coefficients between each two chromatin pairs.

Additionally to define differential chromatin marks among the cell lines, we analyzed the intensities of the regions of ± 1.5 kb from most upstream TSSs for the active and repressive marks (H3K4me3, H3K9/14ac, Pol II, H3K37me3 and H3K9me3) and gene body for the elongation mark (H3K36me3). In this analysis, we used genes with >1 PPM of ChIP-Seq tags in at least one cell line. For enhancers, we calculated the intensities of each representative enhancer region assigned to the genes (within 100 kb upstream of the TSS and gene body). For several cases of differential chromatin marks, qPCR validations were performed (Supplementary Figure S4). Primer sequences were designed by Primer3Plus and provided in Supplementary Table S2D. For other validation studies, ChIP experiments for the selected two datasets were repeated to confirm the reproducibility of the ChIP-Seq data (Supplementary Figure S5). Furthermore, our dataset (H3K4me3 in A549) was compared with data from ENCODE project (Supplementary Figure S6).

Using ChromHMM, which is based on a multivariate hidden Markov model (40), chromatin states were detected and characterized from ChIP-Seq data of the eight chromatin marks. We learned eight chromatin states (41) using ChromHMM and manually annotated them as below: state (i) active promoter; (ii) weak/poised promoter; (iii) strong enhancer; (iv) weak enhancer; (v) transcriptional elongation; (vi) inactive region; (vii) inactive region/heterochromatin and (viii) low/no signal. We also performed ChromHMM for SAEC using the model created by the ChIP-Seq data from the 26 cancer cell lines. For the 26 cancer-related genes, we selected the representative transcript of each gene by manual inspection and also selected the chromatin states that most frequently appeared in the promoter, gene body and enhancers of each gene.

### Analysis of ‘hallmarks of cancer’

To associate the genome, transcriptome and epigenome data of the 26 cell lines with the ‘hallmarks of cancer’ (42), we assigned a total of 2050 genes for the 10 cancer hallmarks. To complement ambiguously annotated genes, we also utilized Gene Ontology (GO) as described in the previous study (43) with manual inspections (Supplementary Table S5A). We further selected the 1840 genes with > 1 RPKM in at least one cell line (Supplementary Table S5B). Genes with mutations in coding sequences (CDS) and splice sites, differential expression, differential DNA methylation and differential chromatin marks (H3K4me3, H3K27me3 and H3K9me3) were counted and assigned to each hallmark.

To characterize common features of cancer cells compared to a normal cell, gene expression levels and intensities of chromatin marks were compared with those of SAEC. For features of gene expression levels, genes with higher or lower expression levels than those of SAEC in at least one cancer cell line were taken as transcriptional aberrations characteristic to cancer under the condition as follows: (i) genes with ≥ 4- or ≤ 1/16-fold RPKM of SAEC in at least one cancer cell line if the genes were transcribed (> 1 RPKM) in SAEC and (ii) > 5 RPKM in at least one cancer cell line if the genes were not transcribed (≤ 1 RPKM) in SAEC. For epigenomic aberrations, genes with higher or lower chromatin marks in at least one cancer cell line were taken under the condition as follows: (i) genes with ≥ 4- or ≤ 1/16-fold ChIP intensities of SAEC in at least one cancer cell line if the genes with > 1 PPM of signal intensities in SAEC and (ii) > 5 PPM in at least one cancer cell line if ≤ 1 PPM in SAEC. A full list of the genes with the detected differential features within the cancer cell lines and compared to the normal cell is also presented in Supplementary Table S5B.

## RESULTS

### Whole-genome sequencing

**Figure 1.**
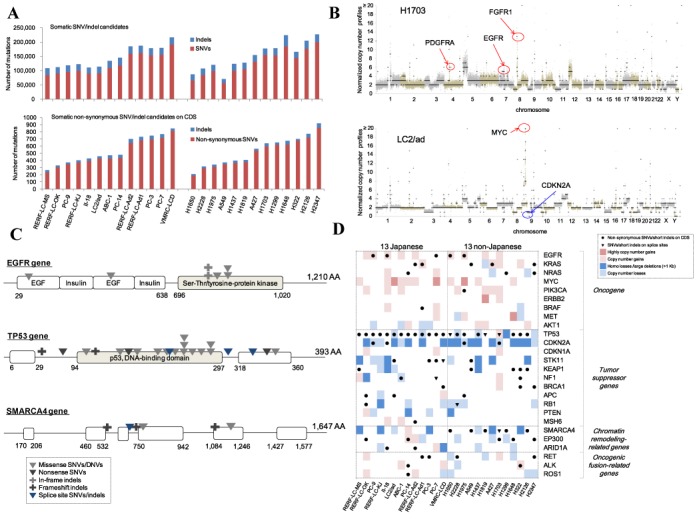
Whole-genome sequencing for genomic aberrations. (**A**) The number of SNVs and indels detected in the 26 cell lines. For each cell line, the number of all somatic mutation candidates and those in the protein-coding regions are shown in the upper and lower panels, respectively. The x-axis is sorted by the origins of the cell lines and the increasing total number of non-synonymous SNVs and indels. (**B**) Examples of copy number information. The normalized copy number profiles of H1703 and LC2/ad are shown in the upper and lower panels, respectively. Examples of genes for which possible CNAs are detected are indicated by arrows (red for amplification and blue for deletion). (**C**) Examples of mutated genes in the 26 cell lines. Mutations identified in the EGFR, TP53 and SMARCA4 genes are shown. Types of mutations are as indicated in the inset. One mutation in the TP53 gene was added by manual inspection. (**D**) Genomic aberration of the selected 26 cancer-related genes. SNVs and indels on the protein-coding regions and splice sites and CNAs are shown.

We generated and analyzed a multilayer-omics catalog of 26 lung adenocarcinoma cell lines (Supplementary Table S1). To determine and characterize somatic mutations in the respective cell lines, we performed whole-genome sequencing. We generated approximately one billion mapped sequences from each cell line, with an average of 33× in coverage and 91% of the genome covered by > 5× in depth. We detected genomic mutations using the pipeline as shown in Supplementary Figure S1. After removing germline mutations registered in public and in-house Japanese databases (96% of the initially called SNVs/indels overlapped with the NCBI dbSNP database) (27), a mean of 149,209 somatic mutation candidates (48 SNVs + indels/Mb) remained for each cell line (Figure 1A and Table 1A). To estimate the frequency of the rates of remaining germline variations, we sequenced and analyzed the normal counterparts derived from B lymphoblasts for three cell lines (H1437, H2126 and H2347). We found that approximately 28% of the somatic mutation candidates were germline and 72% were somatic mutations specific to cancer cells (Supplementary Table S6). Base substitution patterns for SNVs are shown in Supplementary Figure S7. We also detected CNAs and identified averages of 143 copy number gains and 101 losses per cell line in the gene regions (Supplementary Table S7). In addition, we detected a total of 552 genomic rearrangements in the gene regions (Supplementary Table S8).

**Table 1.** The number of SNVs and short indels in the 26 cell lines

	SNVs	Short indels
**(A)**	**Total number of positions (Avg. of the 26 cell lines)**	
Total	12,732,271 (3,302,407)	1,916,622 (453,821)
Germline	10,010,429 (3,177,173)	1,597,810 (429,846)
Somatic candidates	2,721,842 (125,234)	318,812 (23,975)
Genic^a^	892,941 (39,695)	118,268 (8,516)
Upstream (-500 from TSS)	11,796 (551)	2,049 (159)
UTRs	24,902 (1,086)	13 (0.8)
CDS	16,354 (687)	573 (37)
Synonymous	4,505 (188)	***
Non-synonymous	11,849 (499)	***
Splice sites^b^	346 (14)	39 (3)
Intronic and others	839,543 (37,357)	115,594 (8,315)
Intergenic	1,828,901 (85,539)	200,544 (15,459)
		
**(B)**	**Average number of positions in the 26 cell lines**	
Regulatory regions^c^	11,413	2,160
Promoter^d^	2,187	497
Promoter with differential H3K4me3	181	36
Enhancer (H3K4me1)^e^	7,543	1,305
Enhancer with differential H3K4me1	3,163	524
Enhancer (H3K27ac)^e^	5,549	1,006
Enhancer with differential H3K27ac	2,647	465

^a^A total of 19,958 genes were used in this analysis.

^b^The first and last two bases in introns.

^c^Promoters (± 1.5 kb from most upstream TSS) and enhancers assigned to the genes.

^d^A total of 20,598 promoters were used in this analysis.

^e^A total of 683,606 H3K4me1 and 337,545 H3K27ac clusters assigned to the genes were used in this analysis.

Among a total of 3,040,654 somatic SNVs and indels, 33% were identified in the genic or their proximal regions (Table 1A). We found 13,845 mutations within 500 base upstream of the gene regions, 24,915 mutations in the 5′/3′ untranslated regions (UTRs) and 385 mutations in the splice sites (the first and last two bases in introns). Mutations were also detected in potential enhancer regions (see below). For the protein-coding regions in particular, we detected a total of 11,849 non-synonymous SNVs and 573 indels (Figure 1A). An average of 299 mutated genes per cell line was detected with high PolyPhen-2 scores (not benign) (44,45). These numbers are comparable with those obtained from our recent clinical lung adenocarcinoma sequencing analysis, if we assume the estimated frequency of the germline variations are 28% (Supplementary Table S6 and Supplementary Figure S8). These mutations that have been observed in clinical sequencing include those in the EGFR, TP53 and KRAS genes. Also note that, for several cell lines, obvious driver mutations still remained unknown. For our attempt to identify those unknown driver mutations, see Supplementary Figure S9 and Supplementary Table S9. Furthermore, CNAs, as often reported in clinical samples (46,47), were detected in the regions of some cancer-related genes; for example, copy number gains of FGFR1, EGFR and PDGFRA in H1703, amplification of MYC and a homozygous loss of CDKN2A in LC2/ad (Figure 1B).

To further analyze the mutation patterns, we focused on cancer-related genes based on previous lung cancer studies. We selected 26 cancer-related genes with important biological relevance, including nine known oncogenes, eleven tumor-suppressor genes, three chromatin remodeling-related genes and three oncogenic fusion-related genes (1–2,48). We also summarized mutations in 125 genes which have been very recently published as significantly mutated genes in 12 types of cancers by TCGA (49) (Supplementary Figure S10). In the EGFR gene, for example, we detected L858R (in II-18 and H1975) and E746_A750del mutations (in PC-9 and H1650), which are known to be sensitive to the anti-cancer drugs, gefitinib and erlotinib. Furthermore, H1975 was found to harbor the T790M mutation, which is resistant to these drugs (6,50) (Figure 1C, upper panel). We also detected five SNVs in the KRAS gene (including four G12 mutations) and three Q61 mutations in the NRAS gene (51) (Supplementary Figure S11). We observed that the TP53 gene was one of the most frequently mutated genes; 19 cell lines had mutations in its protein-coding region (Figure 1C, middle panel), of which 15 mutations were located in the DNA-binding domain. Notably, we detected splice site mutations in the NF1, STK11, RB1 and TP53 genes, which may cause aberrant splicing in these tumor-suppressor genes (see below). We also detected six mutations (including one splice site mutation) and five large deletions in the SMARCA4 gene which is an epigenetic regulator (2,52–54) (Figure 1C, lower panel). We found that 13 cell lines have large deletions in the CDKN2A gene (48,55–57). A summary of genomic aberrations for the selected 26 genes is shown in Figure 1D.

### RNA-Seq

**Figure 2.**
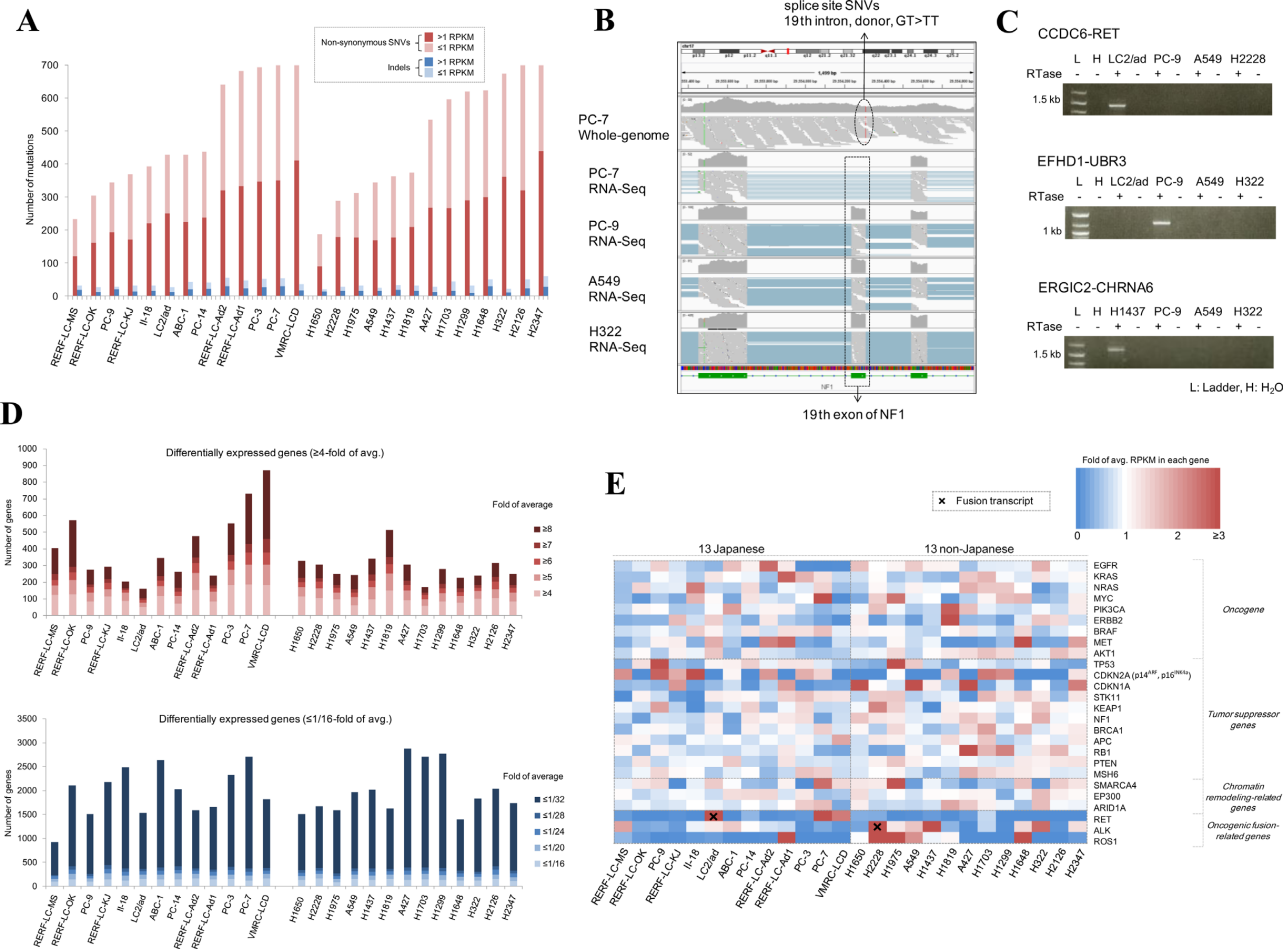
RNA-Seq for transcriptome analyses. (**A**) The number of mutations on expressed genes (> 1 RPKM) and non-expressed genes for each cell line. Non-synonymous SNVs (red) and indels (blue) in the protein coding regions were counted depending on whether their harboring genes are expressed (bright) or not (pale). The x-axis is sorted in the same order as Figure 1A. (**B**) Aberrant splicing events with splice site mutations. For the NF1 gene, IGV visualizes splice site SNVs in whole-genome sequences and the 19th exon skipping in RNA-Seq of PC-7 compared with RNA-Seq of PC-9, A549 and H322 (**C**) Examples of fusion transcripts detected in this study. CCDC6-RET fusion in LC2/ad, EFHD1-UBR3 fusion in PC-9 and ERGIC2-CHRNA6 fusion in H1347 are validated by RT-PCR. (**D**) The numbers of differentially expressed genes are shown for the 26 cell lines (top panel for genes with higher expression and bottom panel for genes with lower expression). (**E**) Gene expression patterns of the 26 cancer-related genes. The heat map represents the fold value against the average expression level in the 26 cell lines. The color key is as shown in the inset.

For the transcriptome analyses, we performed RNA-Seq. Statistics of the RNA-Seq data are shown in Supplementary Table S10. An average of 12,290 genes were expressed at > 1 RPKM (58) in each cell line (also see Supplementary Figure S2 for validation analysis of RNA-Seq). We examined how many of the identified SNVs and indels were located in the transcribed or non-transcribed genes. An average of 254 non-synonymous SNVs and 19 indels, which were approximately half of the total SNVs, were located in the ‘expressed’ genes (Figure 2A). For the genomic mutations located at the splicing sites (Table 1A), we examined whether these SNVs actually affected splicing patterns of the transcripts. As for the cancer-related genes, for example, PC-7 harbored a splice site mutation in the NF1 gene, which is located in the splice donor site of the 19th intron (Figure 2B). The 19th exon of NF1 is skipped in PC-7, demonstrating that this splice site mutation affected the splicing pattern of the NF1 transcript. Transcript consequences of the other splice mutations are shown in Supplementary Figure S12. We also used the RNA-Seq data to detect fusion gene transcripts, which are formed by chromosome rearrangements in cancerous cells. A total of 135 fusion transcript candidates were detected from all the cell lines combined. Several known driver fusion transcripts such as CCDC6-RET (in LC2/ad) were included (10,59–60) (Figure 2C). For the selected cases, RT-PCR validation was conducted (shown in Figure 2C and Supplementary Figure S13). All the previously reported fusion transcripts such as CCDC6-RET and ALK-PTPN3 (in H2228) were computationally re-identified in our study, except for EML4-ALK fusion in H2228 (61) (Supplementary Figure S13), which may have gone undetected by our relatively conservative computational setting due to its low expression level. Most of those aberrant transcripts may not be cancer-drivers but passengers, which have been formed as a consequence of chromosomal aberrations. However, it is worth noting that fusion transcripts can be identified both at the genome and RNA level using this approach.

To dissect gene expression patterns between the cell lines, we selected differentially expressed genes, which showed a higher or lower expression compared to the other cell lines (also see Supplementary Figure S14 for a hierarchical clustering analysis, which represent global expression patterns for each of the cell lines). We tentatively selected genes with ≥ 4- or ≤ 1/16-fold of the average expression levels as ‘differentially expressed’ genes. We detected an average of 352 such higher and 1967 such lower differentially expressed genes in each cell line (Figure 2D). We also examined the expression patterns of the differentially expressed genes in the pathway of ‘lung adenocarcinoma’ (1) (Supplementary Figure S15) and found that each component gene of this pathway showed diverse expression patterns compared to the other pathways. We also investigated the expression patterns for the selected 26 cancer-related genes as shown in Figure 2E. Three cell lines (VMRC-LCD, PC-3 and PC-7) showed almost no expression for the EGFR gene, while H1650 and PC-9, which harbor a driver mutation (E746_A750del), showed higher expression. In contrast, the TP53 and ARID1A genes were expressed at almost the same level (> 1/16 and < 4-fold of the average) throughout the 26 cell lines. Taken together, these results indicate that aberrations in expression patterns, which are distinct from those of genomic aberrations, are also highly diverse among genes and cell types, and such divergence can be explained by complex combinations of contributing regulatory factors ranging from aberrations in the genome and/or in the epigenome.

### Bisulfite sequencing for analyzing DNA methylation

Changes in DNA methylation patterns have been reported in various cancers, which cause aberrant regulation of oncogenes and tumor-suppressor genes (62–65). We performed a target-captured bisulfite sequencing in potential gene regulatory regions including promoters, enhancers and differentially methylated regions (66). For 84 Mb of the bait regions, each dataset had an average depth of 109.7× and 91% were covered by >10× in depth. We also confirmed that the bisulfite conversion rates, which were evaluated as the overall C to T ratio, were 99.2% in all of the 26 cell lines (Supplementary Table S11; detailed statistics are also presented there). We calculated the methylation rate at each CpG site that was covered by ≥ 5 tags and were not overlapping with the detected SNVs and indels. An average of 3,777,270 CpG sites per cell line was considered; 1,273,909 sites were in CpG islands and 2,503,362 sites were in other regions (Supplementary Table S11; also see Supplementary Table S3 and Supplementary Figure S3 for validation study of correct identification of the methylation statuses).

**Figure 3.**
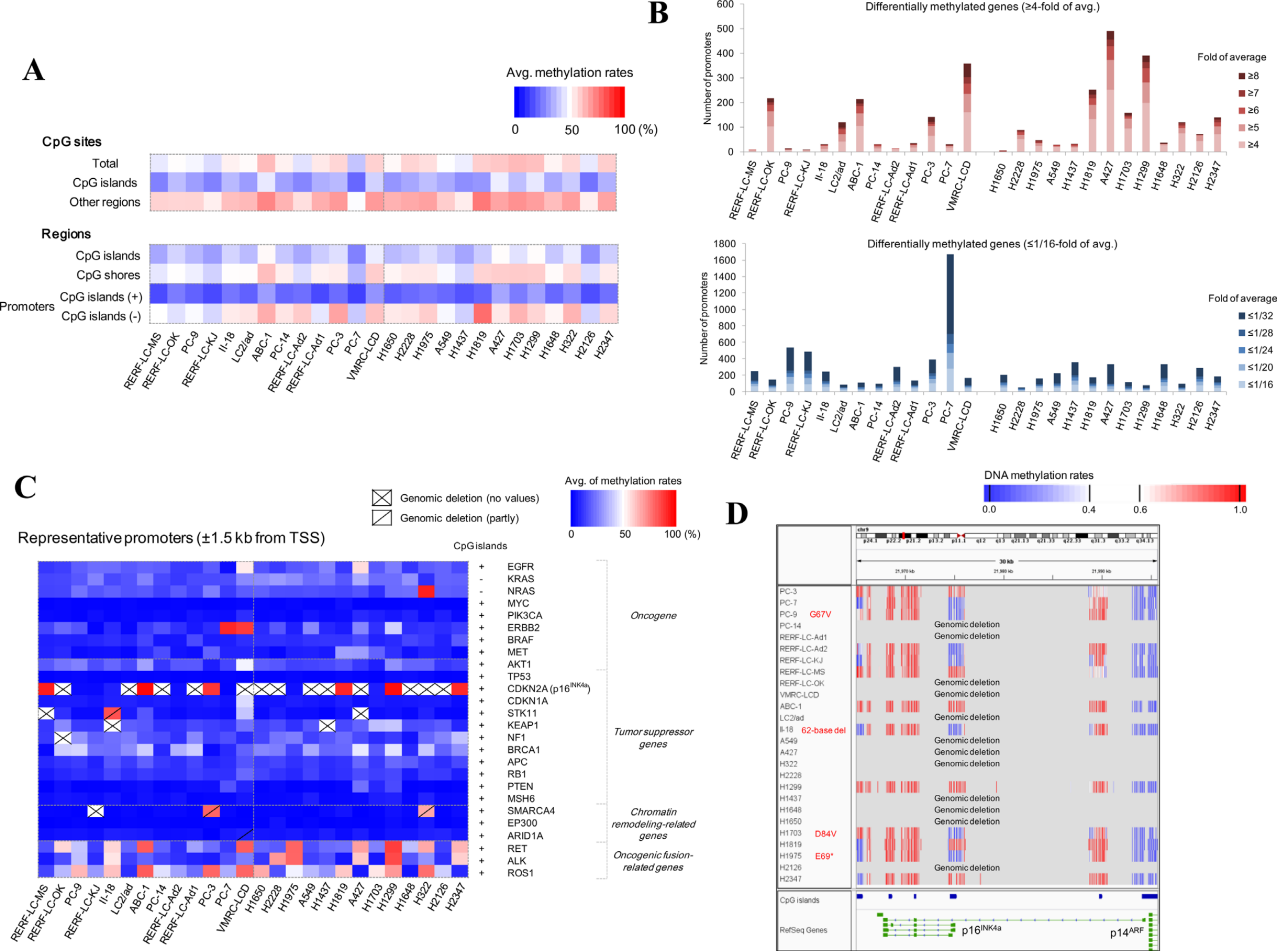
Bisulfite sequencing for analyzing DNA methylation status. (**A**) Summary of DNA methylation in each cell line. Upper panel: average DNA methylation rates are calculated at each CpG site in CpG islands or non-CpG islands to draw the heat map. Lower panel: Results of a similar analysis for the CpG islands, CpG shores and promoters. The color key is shown in the inset. (**B**) The numbers of differentially hyper- (upper panel) or hypo- (lower panel) methylated genes in each of the 26 cell lines. The populations of the genes having the indicated fold changes are separately colored as shown in the insets (**C**) DNA methylation patterns, as indicated by the color key, are shown for the representative promoters of the selected 26 cancer-related genes. Slashes indicate where the genomic deletion was observed. (**D**) DNA methylation of the CDKN2A gene. The degree of methylation at each CpG site (vertical line) is colored as indicated in the inset. Each line represents the information for the indicated cell line. Cell lines for which genomic deletions were observed are also indicated. SNVs and indels detected in p16^INK4a^ were shown in red letters. A gene model is shown in the bottom.

CpG sites in the CpG islands were generally less methylated compared to the other CpG sites (Figure 3A). When we analyzed DNA methylation in the CpG islands and their proximal regions (within 2 kb distance from the CpG islands, so-called ‘CpG shores’) (66), binominal patterns of methylation were observed for the CpG islands; an average of 5914 (23%) were almost fully methylated and 11,901 (46%) were almost non-methylated. In contrast for the CpG shores, moderate methylation was dominant; 64% of the CpG shores showed methylation rates of 10–90%. We also analyzed DNA methylation in the promoters (1.5 kb from TSS). Again, we reconfirmed that the promoters containing CpG islands generally showed lower methylation, consistent with previous papers. However, even for these sites, the degree of methylation was significantly different between the cell lines. This diversity was further enhanced when we considered the methylation rates of the CpG island-negative promoters.

To further examine the patterns of DNA methylation, we conducted a hierarchical clustering analysis (Supplementary Figure S16). We found that H1819 showed the highest DNA methylation, while PC-7 showed the lowest methylation. We also investigated the diversity in the methylation patterns between different cell lines, particularly in the promoter regions. Similar to the RNA-Seq analysis, we searched for differentially methylated genes for which the methylation levels deviated by ≥ 4- or ≤ 1/16-fold from the average of all the cell lines. We detected an average of 118 hyper-methylated and 278 hypo-methylated genes for each cell line (Figure 3B; see Supplementary Figure S17 for examples). In addition, we searched and detected 61 mutations overlapping with the differentially methylated promoters on average for each cell line.

We next examined whether the promoters of the 26 cancer-related genes were differentially methylated (Figure 3C). For the most of the genes, their promoters were non-methylated, indicating that these promoters are active, consistent with the results from the RNA-Seq; however, hyper-methylations were occasionally observed. The promoter of the NRAS gene in H322 was hyper-methylated and the expression level of NRAS was the lowest in this cell line among the 26 cell lines (Supplementary Figure S17). For the CDKN2A (p16^INK4a^) gene, its promoter was hyper-methylated in six cell lines (Figure 3D). For this gene, 13 cell lines originally had no promoter region due to genomic deletions. Additionally, one cell line harbors a 62-base deletion, and three cell lines have non-synonymous SNVs in p16^INK4a^. The CDKN2A gene, for which expression suppressions were reported as major causative events in lung adenocarcinoma (62), DNA methylation should be the dominant cause of the transcriptomic aberrations, following genomic alterations.

### ChIP-Seq for detecting patterns of histone modifications and RNA polymerase II binding profiles

To examine chromatin statuses in the 26 cell lines, we performed ChIP-Seq analysis for seven histone modifications (H3K4me1, H3K4me3, H3K9me3, H3K9/14ac, H3K27ac, H3K27me3 and H3K36me3) and RNA polymerase II (Pol II) (see Supplementary Table S12 for the statistics; see Supplementary Figure S4 for validation analysis). ‘Peaks’ of ChIP-Seq tags were called by MACS2 (38,39) for H3K4me3 and were further associated with the genes, when they were located within 1.5 kb regions of the TSS. On average, H3K4me3 peaks were associated with 12,239 (59%) genes per cell line. In contrast for 2835 (14%) of the total genes, enrichments of repressive markers of H3K27me3 or H3K9me3 were observed in their promoters. For the enhancers, we first associated the MACS2 peaks of H3K4me1 or H3K27ac between the cell lines, considering their mutual overlaps. We identified a total of 847,766 H3K4me1 regions and 426,224 H3K27ac regions in all 26 cell lines combined. These peaks were associated with genes when they are located within 100 kb upstream of the TSS and the gene body. A total of 683,606 marks of H3K4me1 and 337,545 marks of H3K27ac were associated with 19,683 and 18,975 genes, respectively. We further associated these enhancer clusters with genomic mutations. A total of 77,363 SNVs and indels resided in the regions having both H3K4me1 and H3K27ac peaks and 117,246 and 63,478 mutations were located in the regions having only H3K4me1 or H3K27ac peaks, respectively.

**Figure 4.**
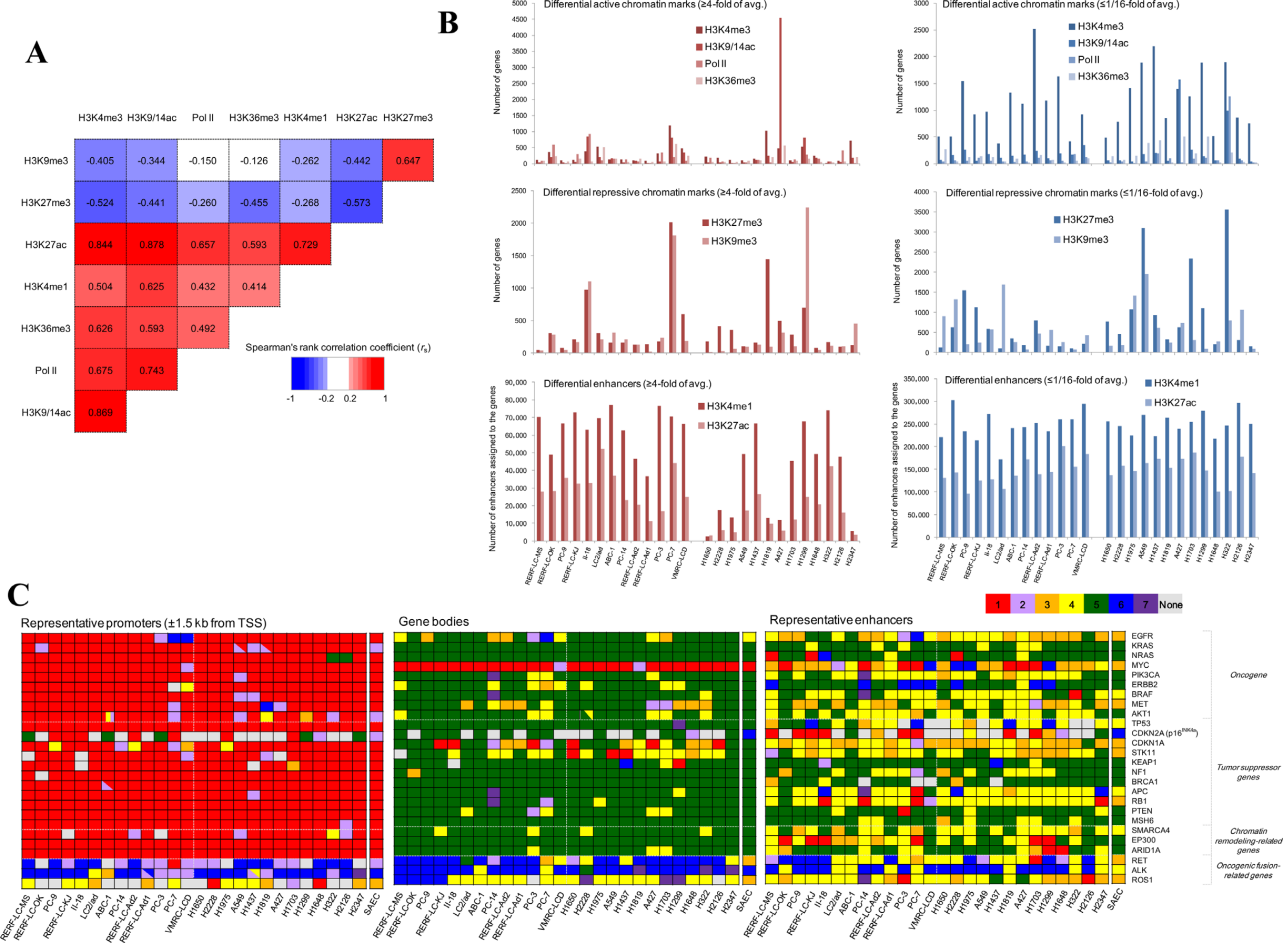
ChIP-Seq for the eight chromatin marks. (**A**) Correlation among the eight chromatin signatures. Spearman's rank correlation coefficients were calculated between the indicated pair of chromatin marks and colored following the color key shown in the inset. Averages of 26 cell lines were used to assign the colors. (**B**) The numbers of differentially utilized chromatin marks for the 26 cell lines. Transcriptional active marks, repressive marks and enhancer marks are represented in the upper, middle and lower panels, respectively. (**C**) Chromatin states based on ChromHMM for the 26 cancer-related genes. ChromHMM maps were drawn for each cell line (see the Materials and Methods section and Supplementary Figure S20). Chromatin states that most frequently appeared in the promoter, gene body and enhancers of each gene are shown in the left, middle and right panels, respectively.

To investigate mutual correlations between the chromatin marks, we calculated the intensities of ChIP-Seq signals in the upstream (up to 1.5 kb from TSS) and in the gene bodies. As shown in Figure 4A, H3K9/14ac and H3K27ac showed the strongest positive correlation (*r*_s_ = 0.878). For the enhancer marks, H3K27ac was also correlated with H3K4me1 (*r*_s_ = 0.729). For the repressive marks, a weak but positive correlation was observed between H3K27me3 and H3K9me3 (*r*_s_ = 0.647). In contrast, active and negative marks had a negative correlation (*r*_s_ = −0.524 for H3K4me3 and H3K27me3). Interestingly, we observed no significant negative correlation between Pol II and H3K9me3, and between H3K36me3 and H3K9me3. Even where positive or negative correlations were observed, the correlations were not always perfect, suggesting there may be several intermediate distinct chromatin statuses even among active or negative statuses (53,67).

We compared the signal intensities of ChIP-Seq tags for each of the chromatin marks. We selected regions that showed ≥ 4- or ≤ 1/16-fold intensities from the average of 26 cell lines (Figure 4B; see Supplementary Figure S18 for an example). In the regions with differential chromatin marks assigned to the genes, we also found a total of 6257 mutations per cell line. In particular, an average of 217 mutations were detected in the promoters with differential H3K4me3 mark and 3687 and 3112 mutations were detected in the enhancers with differential H3K4me1 and H3K27ac marks, respectively (Table 1B). Interestingly, the genes having high H3K9me3 marks were enriched in H1299. For H1299, the DNA methylation pattern was generally high and the number of the hyper-methylated genes was the second largest (Figure 3A, B and Supplementary Figure S16). In contrast, in PC-7, the level of H3K27me3 mark was similarly high in addition to the H3K9me3 mark. Unlike H1299, PC-7 showed lower DNA methylation (Figure 3A and Supplementary Figure S16). Contributions of each of the repressive marks in all 26 cell lines are shown in Supplementary Figure S19. Each cell line may employ distinct expression repression mechanisms, which would not be represented solely by analyses of either DNA methylation or chromatin statuses.

To summarize the eight chromatin marks for the 26 cancer-related genes, we used ChromHMM (40,41) (Figure 4C). We found for the EGFR gene that the patterns of the chromatin signatures were remarkably distinct between cell lines, indicating that each cell line carries an aberration, if any, at a distinct regulatory layer (see Supplementary Figure S20 for the graphic view). For instance, PC-3, PC-7 and VMRC-LCD showed lower expression levels. In PC-7, an active chromatin mark of H3K4me3 was not formed, followed by neither binding signal for Pol II nor H3K36me3. In VMRC-LCD, an H3K4me3 mark was formed, but Pol II was not recruited and H3K36me3 was not formed. In PC-3, H3K4me3 was formed, Pol II was recruited, but an H3K36me3 mark failed to form (Supplementary Figure S21).

### Integrated analysis: genomic, transcriptomic and epigenomic statuses in lung adenocarcinoma cell lines

**Figure 5.**
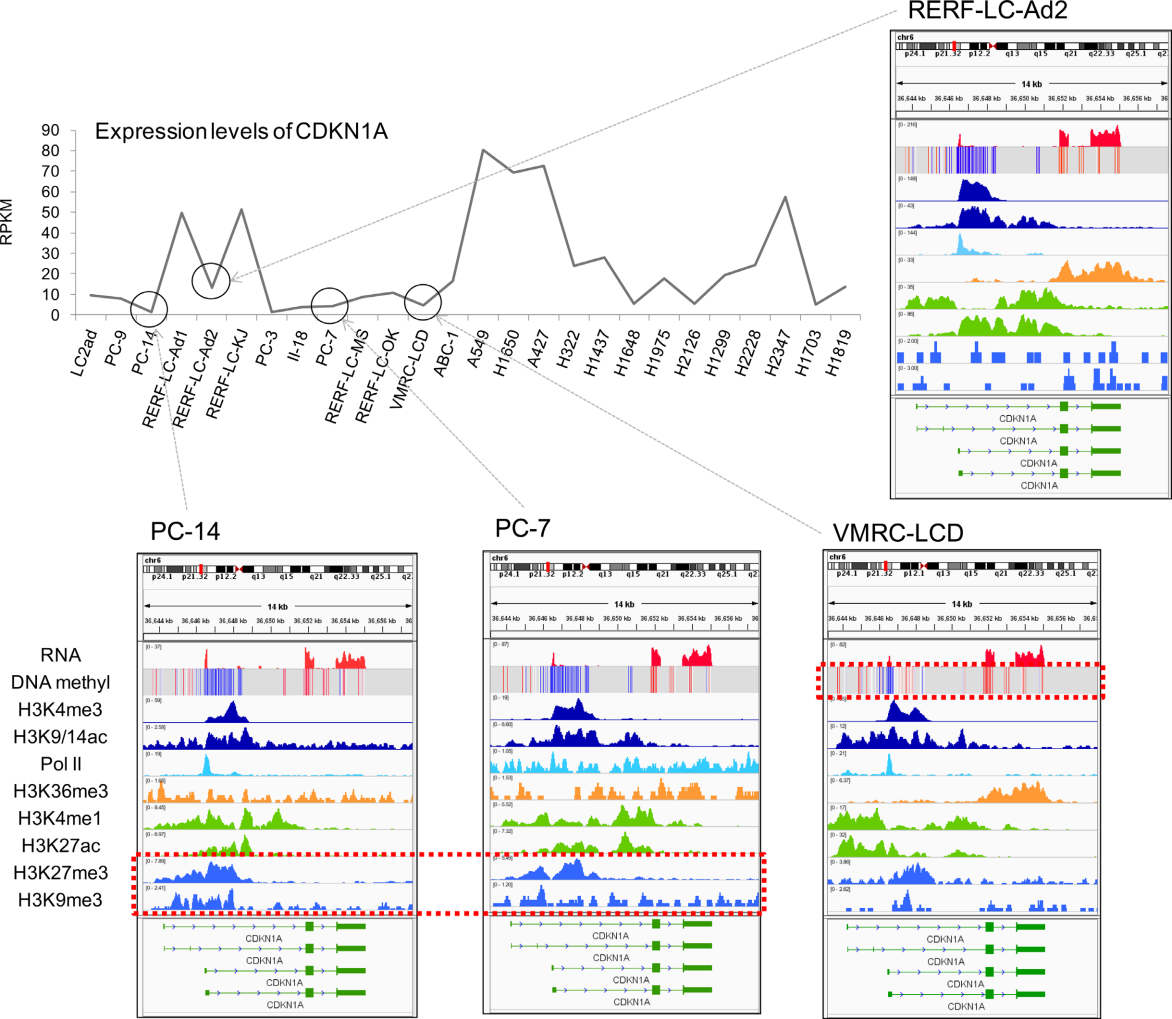
Integrative analysis of multi-omics data. Transcriptomic and epigenomic status of the CDKN1A gene. Expression levels of CDKN1A are shown in the upper graph. RNA-Seq, bisulfite sequencing and ChIP-Seq patterns of CDKN1A are also shown for the four cell lines, indicated in the graph.

By integrating these multi-omics data, we describe which steps of the regulations, namely, genomic alterations, DNA methylation, each step of histone modification or Pol II recruitment, should be impaired to explain eventual irregular expression levels in the respective cell lines. For example, we observed various patterns of gene expression for the STK11 gene, a kinase that plays a pivotal role as a tumor suppressor of lung adenocarcinoma in many cases (1,68), that were completely abolished in three cell lines. Genomic deletions were detected for all of these three cell lines; RERF-LC-MS and A427 lacked the majority of the genic region and II-18 lacked the promoter region (Supplementary Figure S22). In addition, gene expression was repressed in three additional cell lines, H1437, H2126 and RERF-LC-KJ. These three cells have an intact promoter, having the marks of H3K4me3 and Pol II recruited. However, they commonly have large genomic aberrations in the gene body, which may cause the lack of a consequential transcriptional elongation mark of H3K36me3. In another case of the CDKN1A gene, its irregular expression levels were mostly accounted for with epigenomic aberrations (Figure 5) unlike the STK11 gene for which genomic aberrations were the main cause. For example, PC-7 and PC-14 showed higher levels of a repressive mark in its promoter, which may explain its low expression levels in these cell lines. In the VMRC-LCD, the DNA methylation level of its promoter was high. In contrast, for RERF-LC-Ad2, which had a normal expression level of CDKN1A, neither hyper-DNA methylation nor repressive histone marks were observed in the promoter.

We manually inspected for similar diversity in the cancer-related genes. The results of the inferred aberrations are summarized in Table 2 (also see Supplementary Table S13 for Cancer Gene Census (69) genes). In particular, we observed that the genes harboring known driver mutations in the genome, such as the EGFR gene (E746_A750del) in PC-9 and the NRAS gene (Q61K) in H1299, showed retained or even enhanced expression levels, corresponding to their DNA methylation and chromatin patterns. On the other hand, expression levels of TP53 in the 26 cell lines were less diverse than those in the other cancer-related genes, despite that 22 cell lines harbor SNVs or indels in the genome of the TP53 gene. In the TP53 gene, the incidence of genomic aberration was not always coupled with an aberration in the expression level. The regulatory mechanisms that eventually result in an aberration in gene expression in cancer must be diverse among cell lines and may be characteristic to each gene, suggesting the importance in describing the aberration patterns in each regulatory layer of gene expression.

**Table 2.** Genome, transcriptome and epigenome in selected cancer-related genes

Gene	SNVs and indels on CDS or splice sites	SVs^a^	Gene expression (RNA-Seq)	DNA methylation (BS-Seq)	Chromatin signature (ChIP-Seq)
EGFR	5/26	0/26	3/26: no exp	n.s.	3/26: poised/repressive promoter and elongation
KRAS	5/26	0/26	n.s.	n.s.	n.s.
NRAS	3/26	0/26	n.s.	1/26↑	1/26: active and enhancer mark↓
MYC	0/26	3/26: amp	1/26: no exp	n.s.	1/26: active and enhancer mark↓, 1/26: H3K27me3↑
ERBB2	0/26	1/26: amp	1/26↓, 1/26↑	1/26↓, 2/26↑	2/26: H3K4me3↓
MET	0/26	1/26: amp	1/26↑	n.s.	2/26: H3K27me3↑
TP53	22/26	1/26: del	n.s.	n.s.	n.s.
CDKN2A	4/26	13/26: del	13/26: no exp, 5^b^/26↓	5^b^/26↑	n.s.
CDKN1A	0/26	0/26	1/26↓	1/26↑	2/26: poised promoter
STK11	5/26	5/26: del	3/26: no exp	1/26↑	1/26: aberrant elongation mark, 2/26: poised promoter
KEAP1	5/26	2/26: del	2/26: no exp	n.s.	1/26: repressive mark↑
NF1	3/26	1/16: del	n.s.	n.s.	n.s.
SMARCA4	6/26	5/26: del	2/26: no exp	3/26↓	1/26: H3K9me3↑
ARID1A	2/26	1/26: del	n.s.	1/26↓	n.s.
RET	4/26	1/26: fusion	2/26↑, 22/26: no exp	n.s.	1/26: Active promoter, 2/26: H3K36me3↑

^a^SVs: structualvariants.

^b^p16^INK4a^: expression levels examined by CuffLinks.

amp: amplification (normalized copy number ≥ 8); del: deletion (> 1 kb); no exp: ≤ 1 RPKM; n.s.: not significantly differential;

↑: ≥ 4-fold of average; ↓: ≤ 1/16-fold of average.

**Figure 6.**
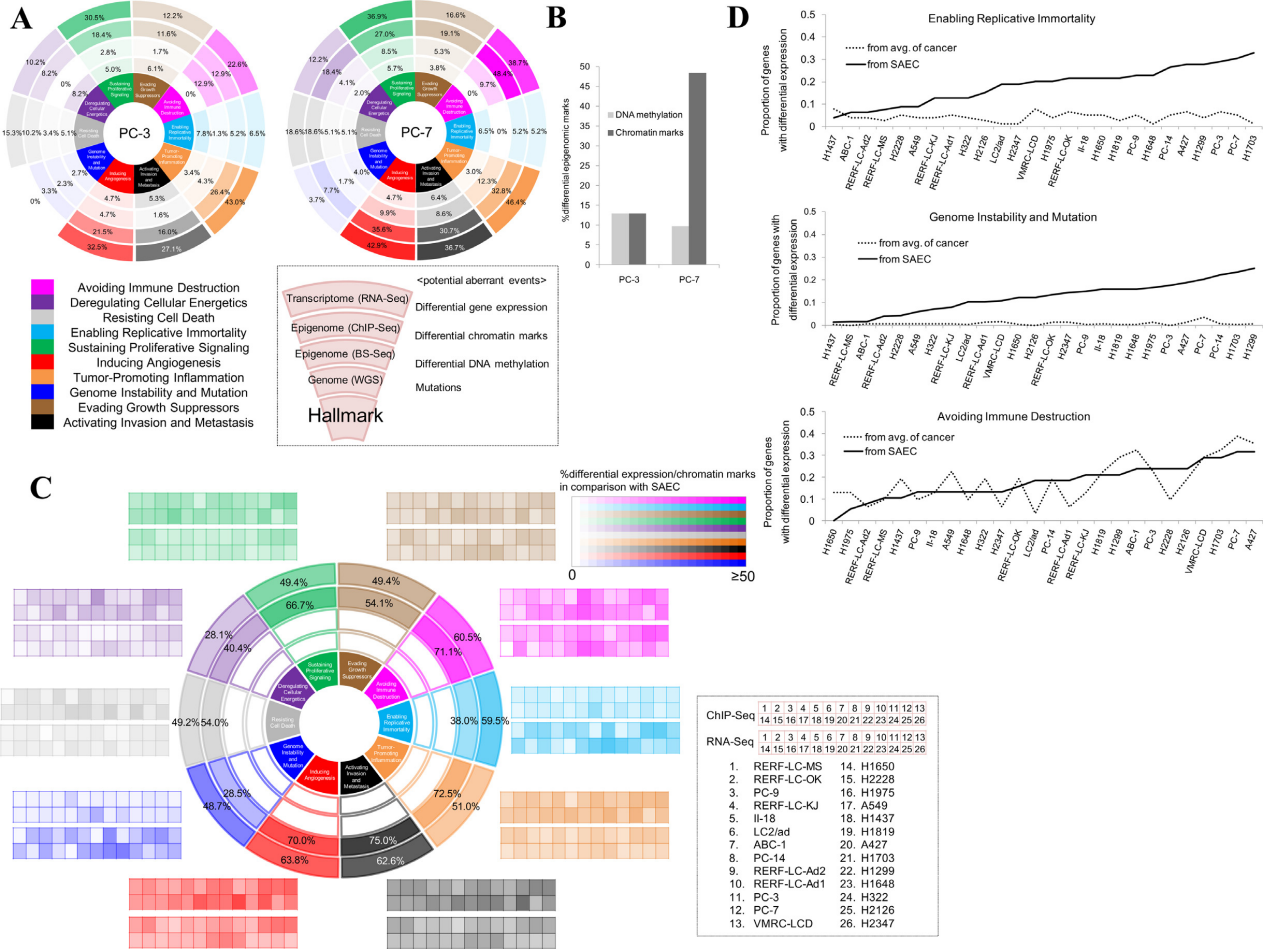
Multi-layerd aberrations in ‘hallmarks of cancer’. (**A**) Potential aberrant events in genome, epigenome and transcriptome in each of 10 hallmarks of cancer. In the cases of PC-3 (left) and PC-7 (right), percentages of genes with mutations, differential expression, differential DNA methylation and differential chromatin marks (H3K4me3, H3K27me3 and H3K9me3) are shown. (**B**) Percentages of genes with differential epigenomic marks in ‘Avoiding Immune Destruction’ for PC-3 and PC-7. (**C**) Aberrant epigenomic and transcriptomic events in cancer cell lines compared to SAEC. Percentages of genes with differential higher or lower expression and chromatin marks were shown for the 26 cell lines. Merged percentages when all of the 26 cell lines are considered are shown. Small square columns in the surrounding margin represent the frequencies in individual cell lines. Color code for the frequency and the order of the cell lines are shown in the right margin. (**D**) Comparison with the variations in the aberrant events (differential features) when compared within cancerous cell lines and deviations from a normal cell, SAEC. Percentages of aberrant features (y-axis) in hallmarks of ‘Enabling Replicative Immortality’ (top), ‘Genome Instability and Mutation’ (middle) and ‘Avoiding Immune Destruction’ (bottom) in the transcriptome layer are shown for the indicated cell lines (x-axis). Solid and broken lines represent the frequencies compared to SAEC and averages of the 26 cancer cell lines, respectively. Cell lines are ordered on the x-axis in order of the increasing frequencies of aberrations in comparison with SAEC (solid line).

To further associate multi-layered features of the 26 cancer cell lines with their features in cancer biology, we employed the concept of ‘hallmarks of cancer’ (42). Many of recent clinical cancer sequencing studies associated the genomic mutation patterns with the impaired functions of a group of genes, each of which represents phenotypic aberrations in cancers. As conducted in previous studies, for each cell line, we associated genomic mutations, differential epigenomic marks and differential gene expression as potential aberrant events with each of the hallmarks. We detected distinct features for each hallmark in multi-omics statuses in 26 cell lines (Supplementary Figure S23A). For example, genes in the hallmarks of ‘Genome Instability and Mutation’ and ‘Enabling Replicative Immortality’ showed little diversity in the transcriptome layer among the cell lines. On the other hand, genes in the ‘Avoiding Immune Destruction’ were differentially represented at the layer of transcriptome among the cancer cell lines, although they harbored only a small number of genomic mutations at the genome layer. These characteristics of the hallmarks allowed us to categorize 26 cell lines conversely (Figure 6A for the cases of PC-3 and PC-7; see Supplementary Figure S23B for the other cell lines). For example, in VMRC-LCD, no differential epigenomic marks were detected in the ‘Deregulating Cellular Energetics’ so that genes in this hallmark could be regulated by different mechanisms comparing with other cell lines. We also found these features are informative to infer how different cell lines achieve the respective hallmarks. For example, for the ‘Avoiding Immune Destruction’, PC-3 utilized DNA methylation to the similar extent with histone modifications, while PC-7 preferentially utilized histone modifications rather than DNA methylation (Figure 6B). Although further in-depth analysis should elucidate those observed characteristic patterns should actually represent distinct phenotypic features of the respective cell lines or biology of their originating cancers, we believe this analysis should be the first step toward that goal.

In order to further characterize which of the identified features in cancer hallmarks are common to the cancer cell lines but not to normal cells, we needed a reference dataset of a normal cell. For this purpose, we newly generated a series of mutli-omics data from a normal SAEC. Similarly to the cases of the other cancerous cell lines, we performed RNA-Seq and ChIP-Seq using SAEC (statistics of the dataset is presented at Supplementary Tables S10 and S12). We used the collected data as an external normal control for transcriptome and epigenome analyses. For the transcriptome analysis, we selected genes which showed higher or lower expression levels in any of the 26 cancer cell lines compared to SAEC and examined which subsets of genes were induced or silenced in given cancer cell line(s). Similarly, for the epigenome analysis, we selected genes with higher or lower ChIP-Seq signal intensities for each chromatin mark. We also performed ChromHMM analysis using the model constructed by the 26 cell lines (Figure 4C).

Based on the collected information, we examined if there are any features common to the cancer cell lines which are distinctive from SAEC regarding the ‘hallmarks of cancer’ (Figure 6C). We found that the induced gene expressions were preferentially observed for the hallmarks of ‘Enabling Replicative Immortality’ and ‘Genome Instability and Mutation’ in cancer cell lines compared to SAEC (Figure 6D). As we have discussed above, gene expression levels in these hallmarks were little diverse among the cancer cell lines. When we also considered SAEC, we found this feature is characteristic to cancer cell lines, but not to a normal cell. In addition, we found that the hallmark of ‘Avoiding Immune Destruction’ is diverse between cancer cell lines but also significantly distinct from a normal cell regarding their epigenomic patterns. Taken together, these results demonstrate the usability of multi-omics data to identify distinct biological features that separate cancer cell lines from a normal cell (also see Supplementary Figure S24 for other examples).

## DISCUSSION

In this study, we generated an integrative multi-omics data of the genome, transcriptome and epigenome of 26 lung adenocarcinoma cell lines. To our knowledge, this is the first dataset, containing a multi-omics data which is collected from the same material, thus can be directly associated.

This is the first study explicitly associating genomic mutations and aberrations in the epigenome and transcriptome with each other. We found that patterns of aberrations were characteristic depending on the cell lines. On the other hand, for the particular genes, we identified several aberrations characteristic depending on the genes, such as deletions in the STK11 gene, chromosome rearrangements in the RET and ALK genes and various types of epigenomic dysregulation in the EGFR, CDKN2A (p16^INK4a^) and CDKN1A genes. These results collectively indicate that various types of aberrations in the regulation of expression as well as mutations involving functional changes in their protein products, such as driver mutations in oncogenes, should play no less important roles in the biology of cancer. Indeed, the first priority should be to investigate further details of transcriptional regulation, starting with the representative cancer-related genes. We believe a cancer ‘regulome’, which is realized by the complex interplay of the genome, epigenome and transcriptome, underlies cancers for which causative molecular events remain unknown.

There are several obvious drawbacks in the present study. First, we could not obtain the normal tissue counterparts for all of the cell lines. Therefore, in this dataset, germline variations have not been completely removed (with the estimated 28% remaining germline variations; Supplementary Figure S8 and Supplementary Table S6). Also, the transcriptomic and epigenomic statuses of each normal tissue counterpart still remain elusive in spite that we used SAEC as a reference control in this study. In addition, there should be significant differences between the cell lines and clinical samples, so that the knowledge obtained from the cell lines should not be directly applied to that obtained from clinical samples.

Nevertheless, it is worth analyzing cancer cell lines for a number of reasons. First, current multi-omics analyses, such as ChIP-Seq and bisulfite sequencing, still require large amounts of starting material, which may not be collected from every clinical sample. Indeed, in most of the clinical cancers, molecular mechanisms to serve as a driver still remain elusive, in spite of rapidly growing repertoires of genomic mutations. It is supposed that the ‘regulatory’ aberration in cancers may be no less important as genomic drivers, though such drivers could not be directly identified solely on the analysis of genomic mutations. Indeed, our analysis on hallmarks of cancer, based on multi-omics data, shed the first light on how disruptions in regulatory elements will realize deviated gene expression programs in cancers. Second, once any indication is obtained, it is inevitable to use cell lines as an *in vitro* model system to conduct any functional validation. For genetic disruptions or drug administrations, a surrogate of the clinical tissues which has the same mutation/expression aberration patterns should be needed. Perhaps the most important advantage of the generated multi-omics catalog for the clinical usage is that appropriate cell lines can be selected for drug test both for an *ab initio* massive drug screening and for personalized medicine. All of the multi-omics data obtained in the present study has been made public and is freely available from our database (http://dbtss.hgc.jp/). Visual inspection for each gene is also enabled. We believe in the importance of the multi-omics data generated in this study to expedite clinical cancer genomic studies in the future.

## SUPPLEMENTARY DATA

Supplementary Data are available at NAR Online.

SUPPLEMENTARY DATA
