# Supplementary material for: Aberrant transcriptional regulations in cancers: genome, transcriptome and epigenome analysis of lung adenocarcinoma cell lines
Source: Nucleic Acids Res. 2014 Nov 6;42(22):13557–72. doi: 10.1093/nar/gku885 (PMC4267666; doi:10.1093/nar/gku885)
Supplement: SUPPLEMENTARY DATA [file supp_42_22_13557__index.html]

Aberrant transcriptional regulations in cancers: genome, transcriptome and epigenome analysis of lung adenocarcinoma cell lines — Aberrant transcriptional regulations in cancers: genome, transcriptome and epigenome analysis of lung adenocarcinoma cell lines — SUPPLEMENTARY DATA 

# Aberrant transcriptional regulations in cancers: genome, transcriptome and epigenome analysis of lung adenocarcinoma cell lines

## SUPPLEMENTARY DATA

**Files in this Data Supplement:**

- SUPPLEMENTARY DATA
