# Supplementary material for: Aberrant transcriptional regulations in cancers: genome, transcriptome and epigenome analysis of lung adenocarcinoma cell lines
Source: Nucleic Acids Res. 2014 Nov 6;42(22):13557–72. doi: 10.1093/nar/gku885 (PMC4267666; doi:10.1093/nar/gku885)
Supplement: SUPPLEMENTARY DATA [file supp_gku885_nar-02466-x-2014-File008.zip › Supplementary_Data(1).pdf]

Aberrant transcriptional regulations in cancers:
genome, transcriptome and epigenome analysis of lung adenocarcinoma cell lines

Ayako Suzuki, Hideki Makinoshima, Hiroyuki Wakaguri, Hiroyasu Esumi, Sumio Sugano, Takashi Kohno, Katsuya Tsuchihara, Yutaka Suzuki

SUPPLEMENTARY MATERIALS

SUPPLEMENTARY FIGURES S1 - S24 (p. 2 - pp. 32)
SUPPLEMENTARY TABLES S1, **S6 - S8**, **S10 - S 12** (pp. 33 - pp. 39)
REFERENCE (pp. 40 - pp. 41)

Supplementary Table S2 - S5, S9 and S13 are provided in separate Excel files.

SUPPLEMENTARY FIGURES

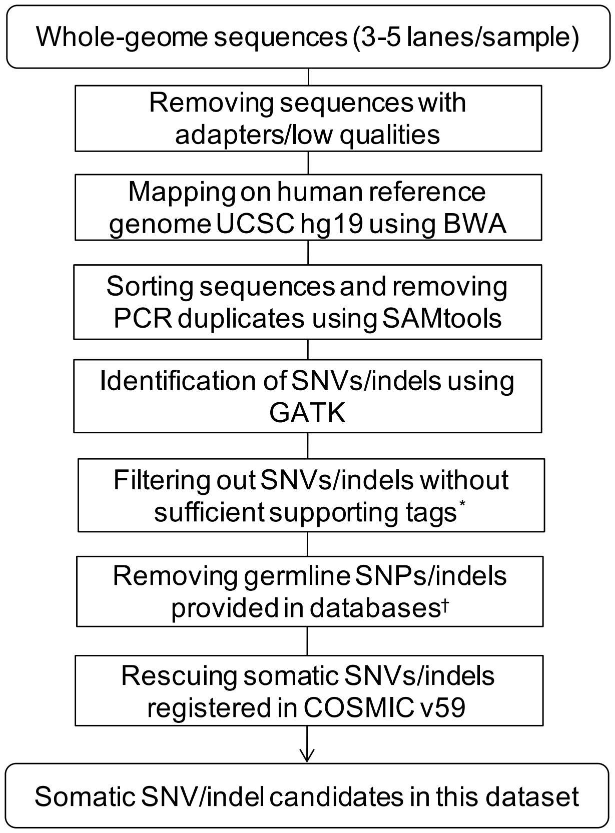

Supplementary Figure S1. The workflow to detect SNVs and short indels using whole-genome sequencing data.

The paired-end sequences were mapped to the human reference genome using the Burrows-Wheeler Aligner (BWA) (1). PCR duplicates were discarded using SAMtools (2). SNVs and indels were identified using the Genome Analysis Toolkit (GATK) Unified Genotyper or Somatic Indel Detecter (3,4), respectively. After filtering SNVs and indels under certain conditions, germline mutations registered in the public databases and in-house catalogues of Japanese normal variations were removed, and somatic mutations in COSMIC (5,6) were rescued.

*Sufficient supporting tags: for SNVs, variant tags ≥ 4; for indels, variant tags ≥ 4, variant tags (Fwd) ≥ 1 and variant tags (Rev) ≥ 1.

†NCBI dbSNP build 137 (7), Exome Sequencing Project (ESP6500SI-V2) (8) (AF > 0.1%), the 1000 Genomes Project (9) (phase1_v3, downloaded on 2013.10.10) (AF > 0.1%) and in-house Japanese SNP data from 145 Japanese normal tissues.

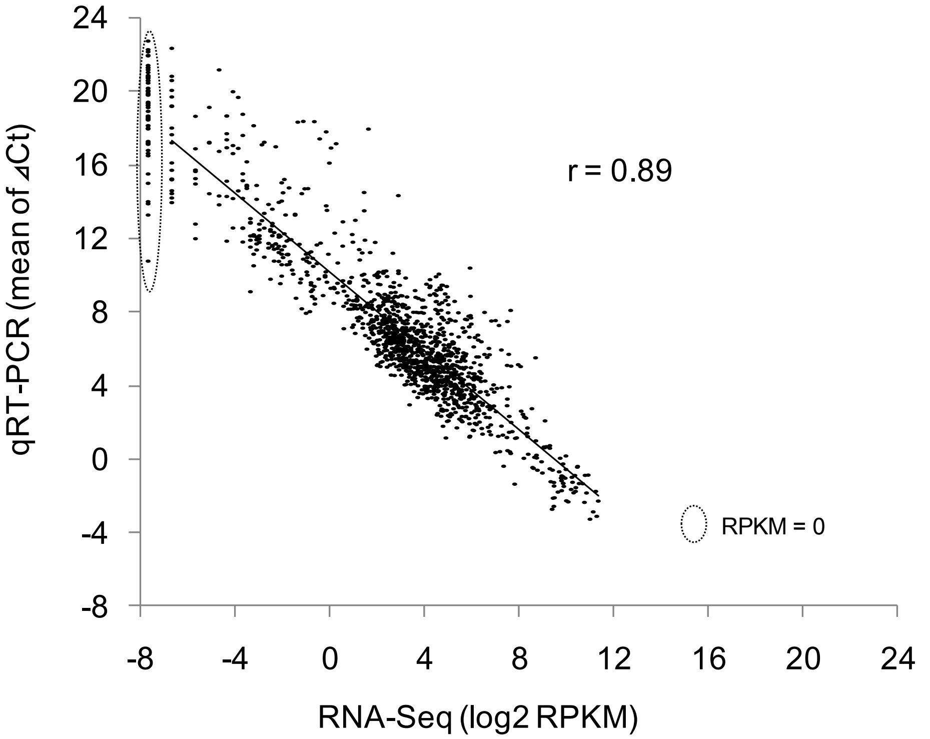

Supplementary Figure S2. qRT-PCR for validations of RNA-Seq data.

For validation of expression abundances, qRT-PCR validations were performed for 52 genes in the 26 cell lines (n = 3). 1,352 plots of expression levels calculated from RNA-Seq (log2 RPKM) and qRT-PCR (⊿Ct = Ct - Ct $_{\text{GAPDH}}$) were shown in the graph. "Undetermined" Ct was taken as 40. The correlation coefficient was represented in the inset. 54 plots (RPKM = 0) were not used to calculate the correlation coefficient. The results of RNA-Seq and qRT-PCR showed strong positive correlation (r = 0.89). Primer sequences were provided in **Supplementary Table S2A**.

A

TTC23 in H2126

Direct sequencing

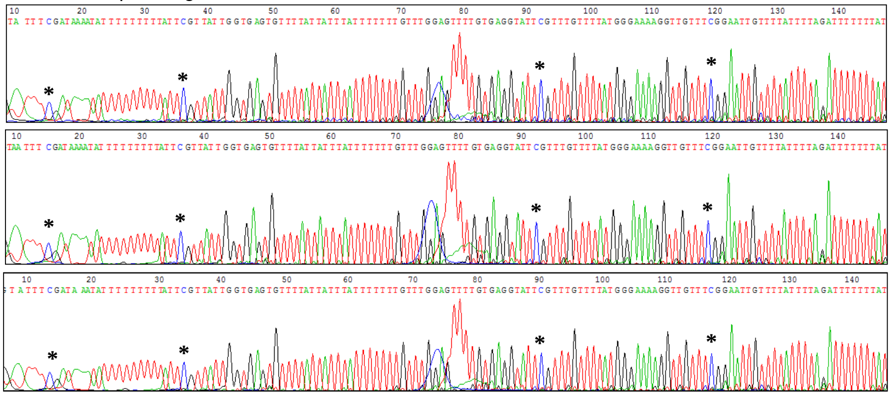

TA cloning

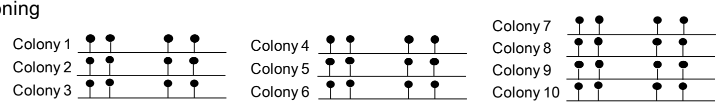

Methylation rate (%)

	chr15:99,789,855	chr15:99,789,833	chr15:99,789,777	chr15:99,789,750
Bisulfite sequencing	92.9	95.4	98.4	97.4
Direct sequencing (avg.)	100	100	100	100
TA cloning	100	100	100	100

B

TTC23 in H2347

Direct sequencing

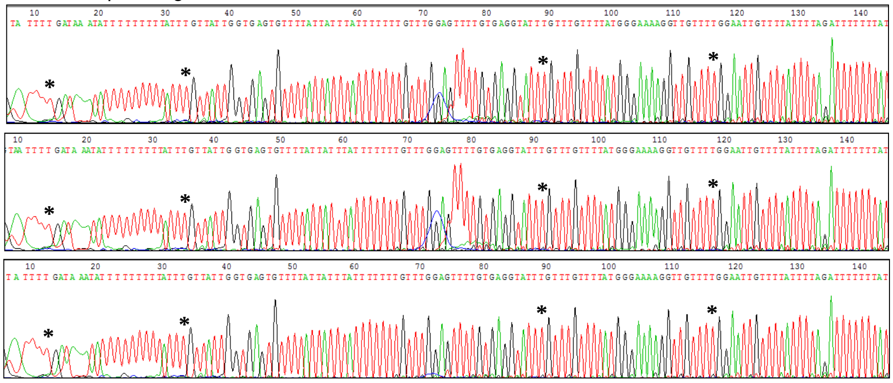

TA cloning

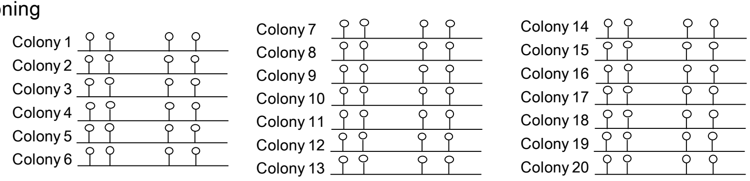

Methylation rate (%)

	chr15:99,789,855	chr15:99,789,833	chr15:99,789,777	chr15:99,789,750
Bisulfite sequencing	0	0	0.43	0
Direct sequencing (avg.)	0	0	0	0
TA cloning	0	0	0	0

C

TTC23 in H2228

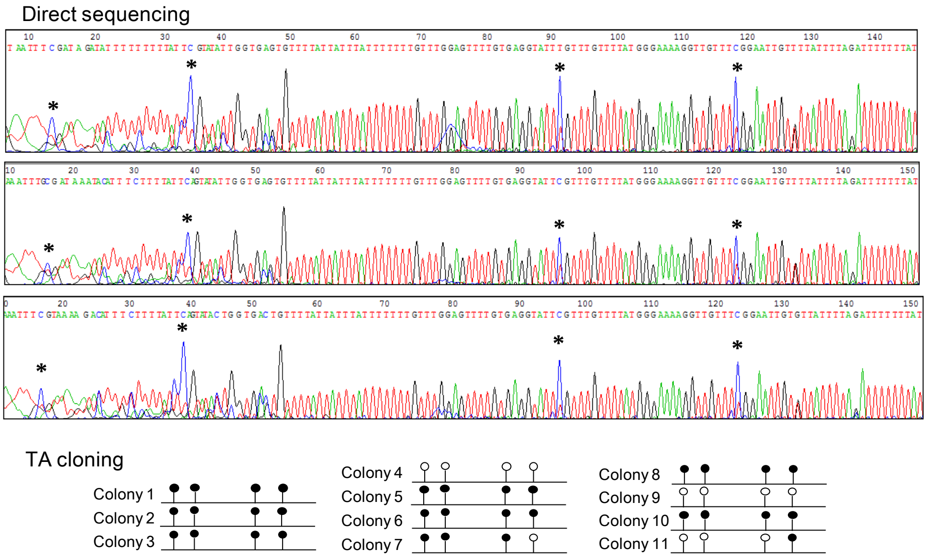

Methylation rate (%)

	chr15:99,789,855	chr15:99,789,833	chr15:99,789,777	chr15:99,789,750
Bisulfite sequencing	57.9	61.9	68	62
Direct sequencing (avg.)	64.9	77.3	74.7	74
TA cloning	72.7	72.7	72.7	72.7

Supplementary Figure S3. Sanger sequencing for validation of bisulfite sequencing data.

The results of direct Sanger sequencing (n = 3) and TA cloning (≥ 10 colonies) for four CpG sites in TTC23 gene were shown. This region showed differential DNA methylation in the three cell lines H2126 (**A**), H2347 (**B**) and H2228 (**C**). PCR primers used for the assays are provided in **Supplementary Table S2C**.

<u>**Summary of validation analysis**</u>: For validation study of the bisulfite sequencing data, we performed direct Sanger sequencing for 30 assays with triplicates for 13 genes. Among them, we further performed TA cloning for 12 assays for seven genes. We used the data only when the sequence data for ≥10 colonies for each sample was obtained. In total, data of the methylation rates was obtained for 149 CpG sites. We compared the results with the bifulfite sequencing data. We found that methylation rates for the most of the CpG sites (83%, 124/149) were correctly represented in Sanger sequencing data (**Supplementary Table S3**).

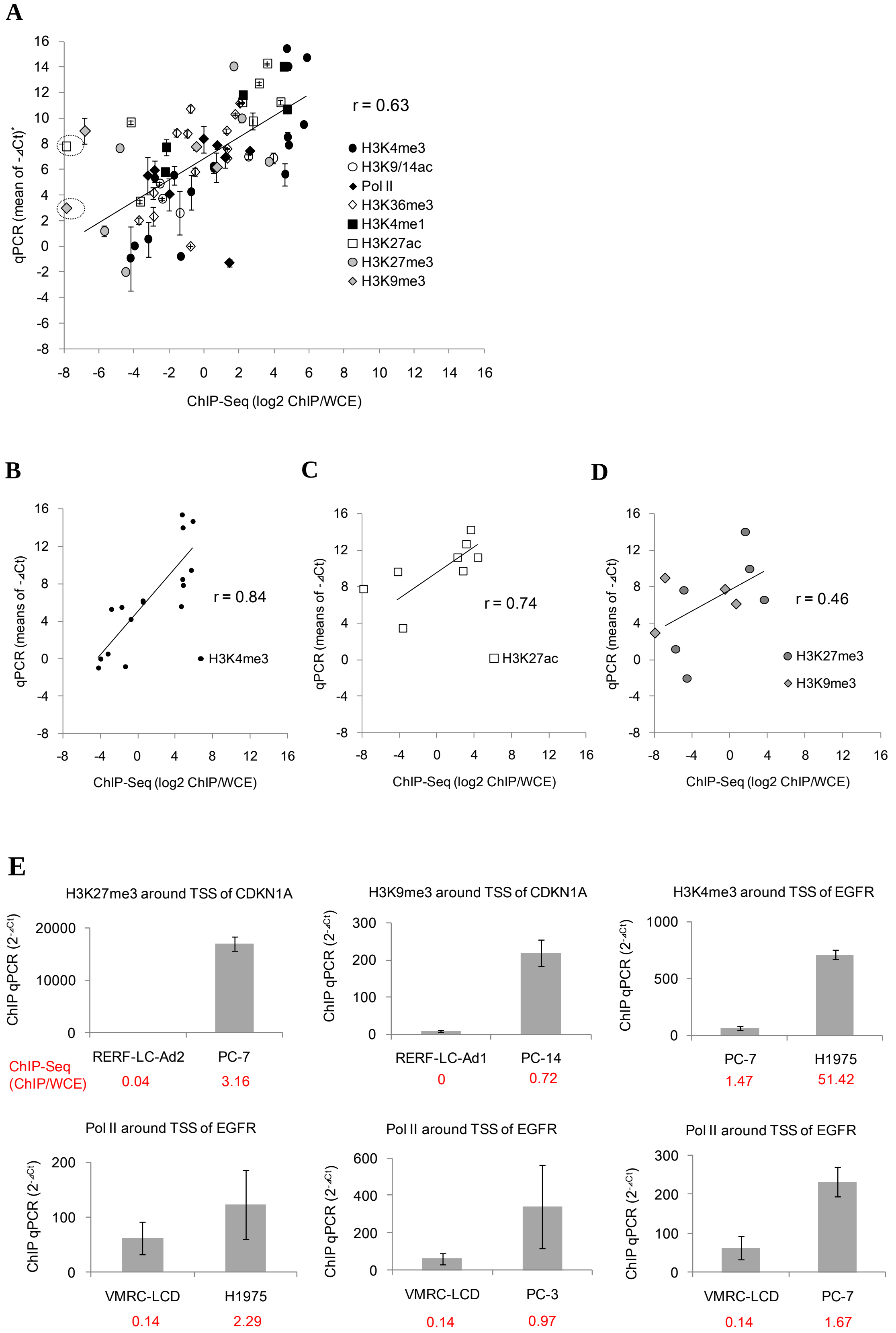
A
r = 0.63
H3K4me3
H3K9/14ac
Pol II
H3K36me3
H3K4me1
H3K27ac
H3K27me3
H3K9me3
qPCR (mean of -⊿Ct)*
ChIP-Seq (log2 ChIP/WCE)
B
r = 0.84
H3K4me3
qPCR (means of -⊿Ct)
ChIP-Seq (log2 ChIP/WCE)
C
r = 0.74
H3K27ac
qPCR (means of -⊿Ct)
ChIP-Seq (log2 ChIP/WCE)
D
r = 0.46
H3K27me3
H3K9me3
qPCR (means of -⊿Ct)
ChIP-Seq (log2 ChIP/WCE)
E
H3K27me3 around TSS of CDKN1A
ChIP qPCR (2-⊿Ct)
RERF-LC-Ad2
PC-7
ChIP-Seq (ChIP/WCE)
0.04
3.16
H3K9me3 around TSS of CDKN1A
RERF-LC-Ad1
PC-14
0
0.72
H3K4me3 around TSS of EGFR
PC-7
H1975
1.47
51.42
Pol II around TSS of EGFR
VMRC-LCD
H1975
0.14
2.29
Pol II around TSS of EGFR
VMRC-LCD
PC-3
0.14
0.97
Pol II around TSS of EGFR
VMRC-LCD
PC-7
0.14
1.67

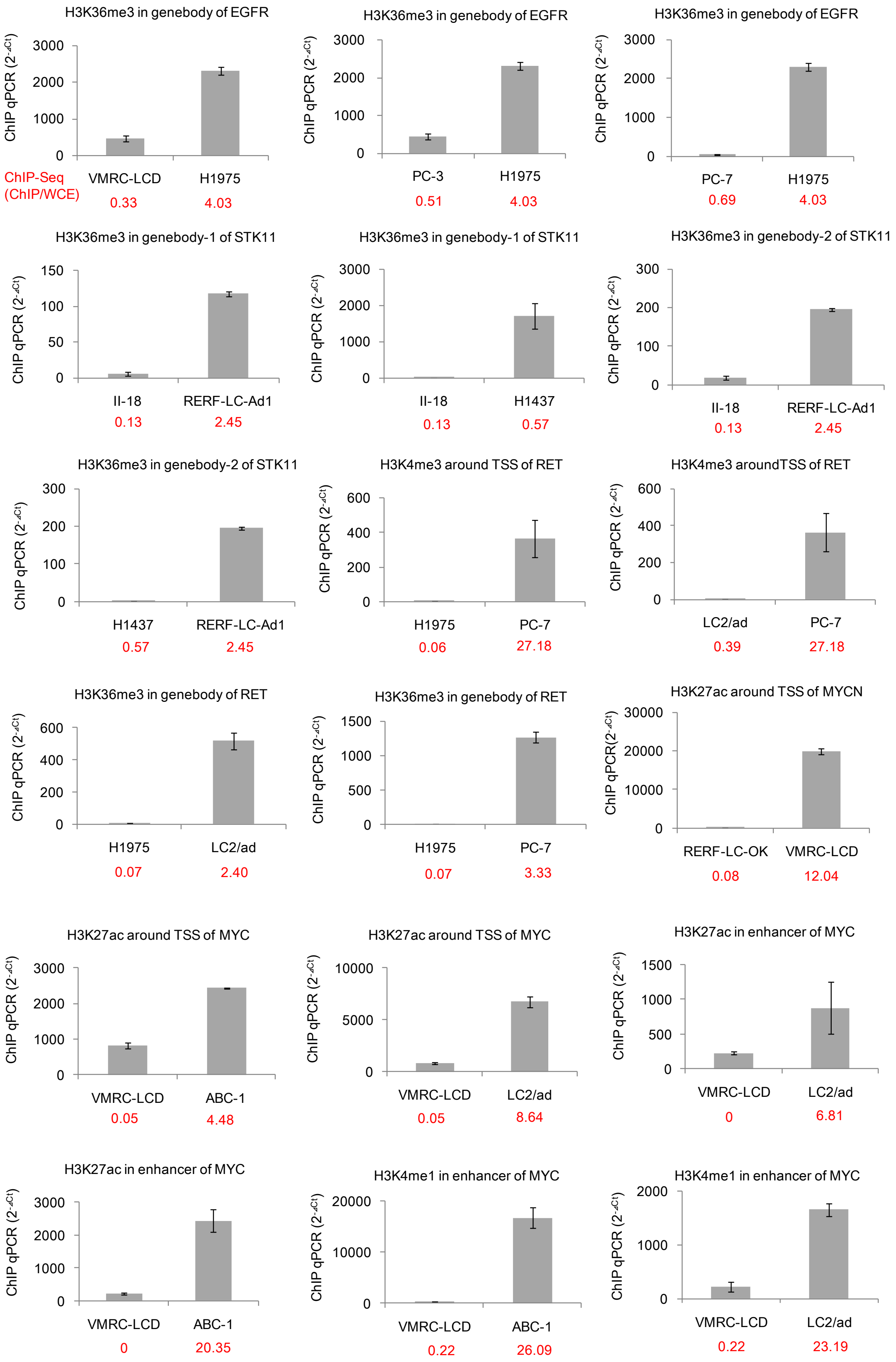

ChIP qPCR ($2^{-\Delta Ct}$)
ChIP-Seq (ChIP/WCE)
H3K36me3 in genebody of EGFR
VMRC-LCD 0.33
H1975 4.03
H3K36me3 in genebody of EGFR
PC-3 0.51
H1975 4.03
H3K36me3 in genebody of EGFR
PC-7 0.69
H1975 4.03
H3K36me3 in genebody-1 of STK11
II-18 0.13
RERF-LC-Ad1 2.45
H3K36me3 in genebody-1 of STK11
II-18 0.13
H1437 0.57
H3K36me3 in genebody-2 of STK11
II-18 0.13
RERF-LC-Ad1 2.45
H3K36me3 in genebody-2 of STK11
H1437 0.57
RERF-LC-Ad1 2.45
H3K4me3 around TSS of RET
H1975 0.06
PC-7 27.18
H3K4me3 aroundTSS of RET
LC2/ad 0.39
PC-7 27.18
H3K36me3 in genebody of RET
H1975 0.07
LC2/ad 2.40
H3K36me3 in genebody of RET
H1975 0.07
PC-7 3.33
H3K27ac around TSS of MYCN
RERF-LC-OK 0.08
VMRC-LCD 12.04
H3K27ac around TSS of MYC
VMRC-LCD 0.05
ABC-1 4.48
H3K27ac around TSS of MYC
VMRC-LCD 0.05
LC2/ad 8.64
H3K27ac in enhancer of MYC
VMRC-LCD 0
LC2/ad 6.81
H3K27ac in enhancer of MYC
VMRC-LCD 0
ABC-1 20.35
H3K4me1 in enhancer of MYC
VMRC-LCD 0.22
ABC-1 26.09
H3K4me1 in enhancer of MYC
VMRC-LCD 0.22
LC2/ad 23.19

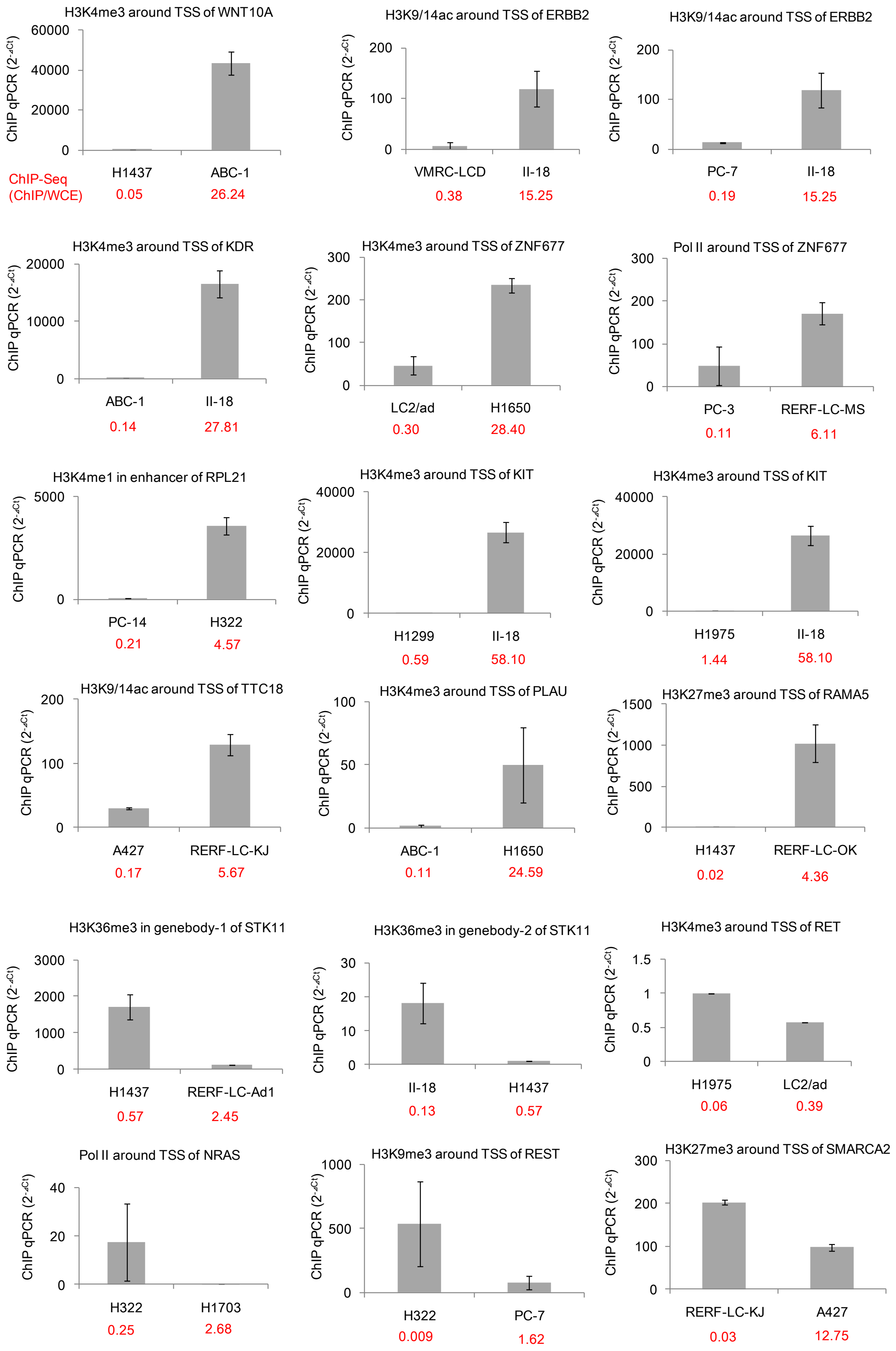
H3K4me3 around TSS of WNT10A
ChIP qPCR (2-ΔCt)
ChIP-Seq (ChIP/WCE)
H1437 0.05
ABC-1 26.24
H3K9/14ac around TSS of ERBB2
VMRC-LCD 0.38
II-18 15.25
H3K9/14ac around TSS of ERBB2
PC-7 0.19
II-18 15.25
H3K4me3 around TSS of KDR
ABC-1 0.14
II-18 27.81
H3K4me3 around TSS of ZNF677
LC2/ad 0.30
H1650 28.40
Pol II around TSS of ZNF677
PC-3 0.11
RERF-LC-MS 6.11
H3K4me1 in enhancer of RPL21
PC-14 0.21
H322 4.57
H3K4me3 around TSS of KIT
H1299 0.59
II-18 58.10
H3K4me3 around TSS of KIT
H1975 1.44
II-18 58.10
H3K9/14ac around TSS of TTC18
A427 0.17
RERF-LC-KJ 5.67
H3K4me3 around TSS of PLAU
ABC-1 0.11
H1650 24.59
H3K27me3 around TSS of RAMA5
H1437 0.02
RERF-LC-OK 4.36
H3K36me3 in genebody-1 of STK11
H1437 0.57
RERF-LC-Ad1 2.45
H3K36me3 in genebody-2 of STK11
II-18 0.13
H1437 0.57
H3K4me3 around TSS of RET
H1975 0.06
LC2/ad 0.39
Pol II around TSS of NRAS
H322 0.25
H1703 2.68
H3K9me3 around TSS of REST
H322 0.009
PC-7 1.62
H3K27me3 around TSS of SMARCA2
RERF-LC-KJ 0.03
A427 12.75

Supplementary Figure S4. qPCR for validations of ChIP-Seq data.

qPCR validations for intensities of chromatin marks from ChIP-Seq data. (**A**) For the strict quantitative evaluation, the results of 65 qPCR assays were plotted in comparison with the ChIP-Seq intensities (n = 3, $\triangle Ct = Ct_{ChIP} - Ct_{WCE}$). Two plots (ChIP/WCE = 0) were not used to calculate the correlation coefficient. Error bars represented ±2 sd of the mean. "Undetermined" Ct was taken as 40. 100 pg of ChIP or WCE DNA were used in this analysis. The results of ChIP-Seq and qPCR were moderately correlated (r = 0.63). (**B-D**) The results of each histone modification. The results of H3K4me3 (**B**) and H3K27ac (**C**) showed strong positive correlations (r = 0.84 and 0.74, respectively), while the correlation of the repressive marks (**D**) were not quantitative (r = 0.46 in the case of H3K27me3 and H3K9me3). This lack of strong qualitative correlation may be derived from the nature of their broad peak patterns in ChIP assays, thus, may be difficult to be represented by a given set of qPCR primers. (**E**) Comparison of the results between qPCR assays and ChIP-Seq in 42 pairs of the cases for which we observed more than four-fold difference in ChIP-Seq intensities. ChIP-Seq intensities were shown in red letter. 85.7% of the pairs of the qPCR results (36/42) were consistent with the patterns of the ChIP-Seq, supporting differential histone modifications. Primer sequences were provided in **Supplementary Table S2D**.

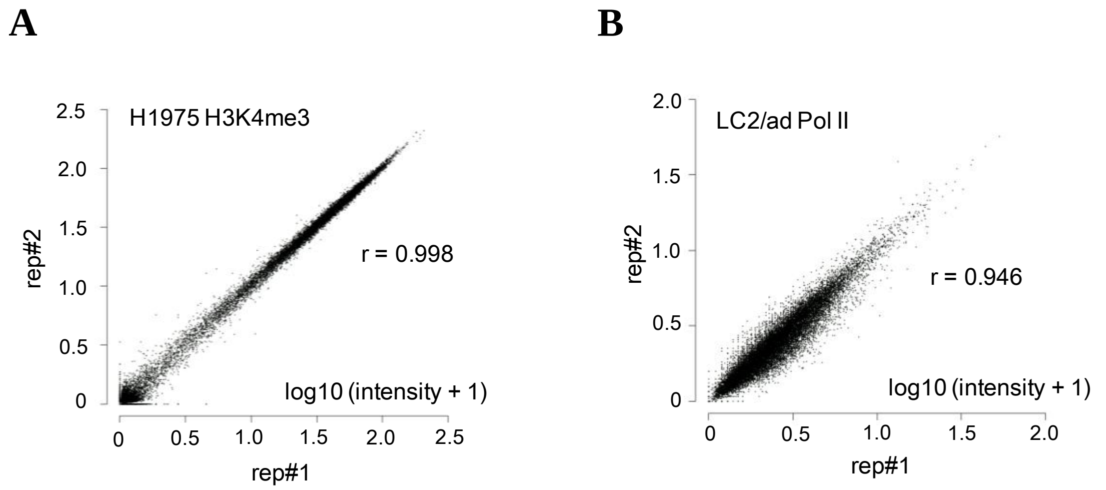

Supplementary Figure S5. Replicates of ChIP-Seq data.

ChIP experiments for two datasets (H3K4me3 in H1975 (**A**) and Pol II in LC2/ad (**B**)) were repeated to confirm the reproducibility of ChIP-Seq data. For 20,598 genes, intensities of ChIP-Seq data ([ChIP PPM] / [WCE PPM], ±1.5 kb from TSS) were calculated and compared. The intensities were adjusted by +1 and log10 transformed for the graphs and Pearson's correlation coefficients. The intensities of ChIP-Seq showed strong positive correlation (r = 0.998 for H3K4me3 in H1975 and r = 0.946 for Pol II in LC2/ad).

A

Number of genes overlapping* with narrow peaks

	Our dataset	ENCODE rep#1	ENCODE rep#2
A549 H3K4me3	11,898	13,424	13,375

11,820 (87.5%)

11,807 (87.7%)

13,262 (98.0%)

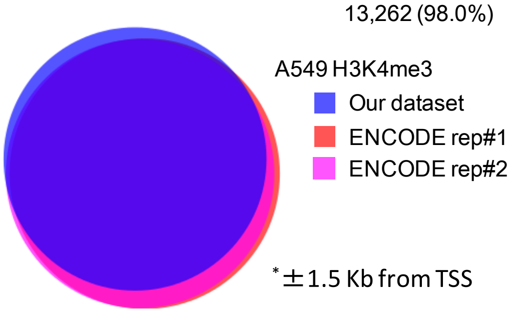

*±1.5 Kb from TSS

B

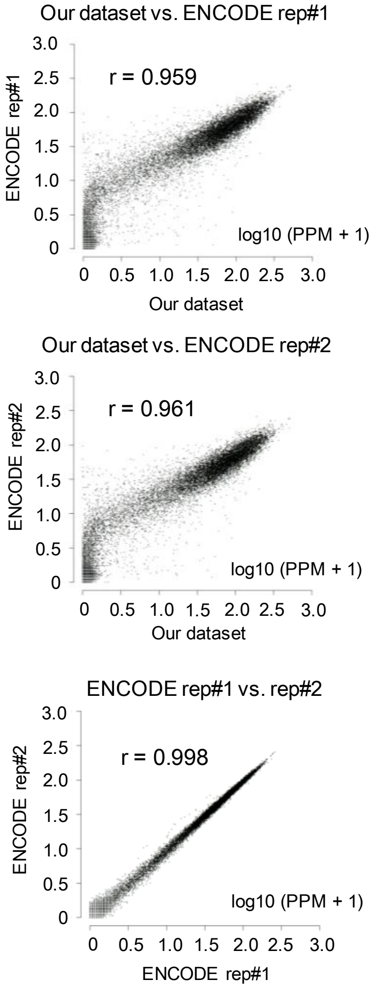

Supplementary Figure S6. Comparison of ChIP-Seq data between our dataset and ENCODE project.

The ChIP-Seq dataset for H3K4me3 of A549 was compared with data provided from ENCODE project (wgEncodeEH001905 and wgEncodeEH001904). (**A**) Overlaps of narrow peaks. Most of peaks were overlapped among datasets. (**B**) Correlation of signal intensities (PPM, ±1.5 kb from TSS). PPMs were adjusted by +1 and log10 transformed for the graphs and Pearson's correlation coefficients. Signal intensities among datasets showed strong positive correlation.

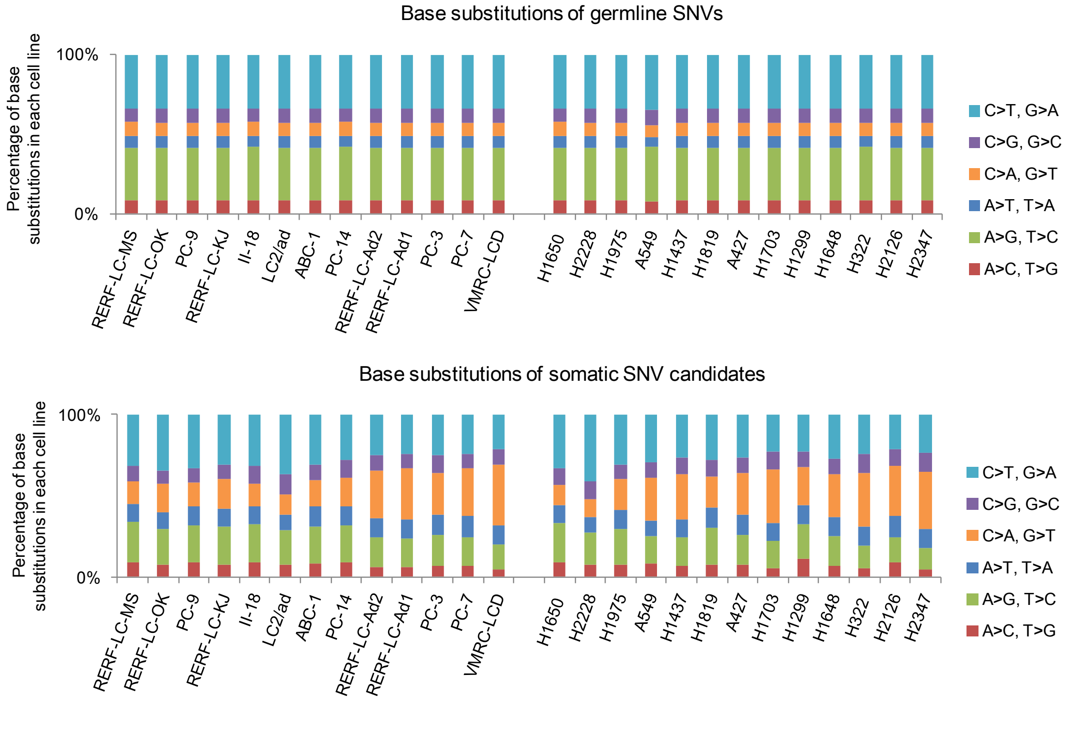

Supplementary Figure S7. Base substitution patterns of the 26 cell lines.

The base substitution patterns of germline variants and somatic SNV candidates are shown in upper and lower panel, respectively.

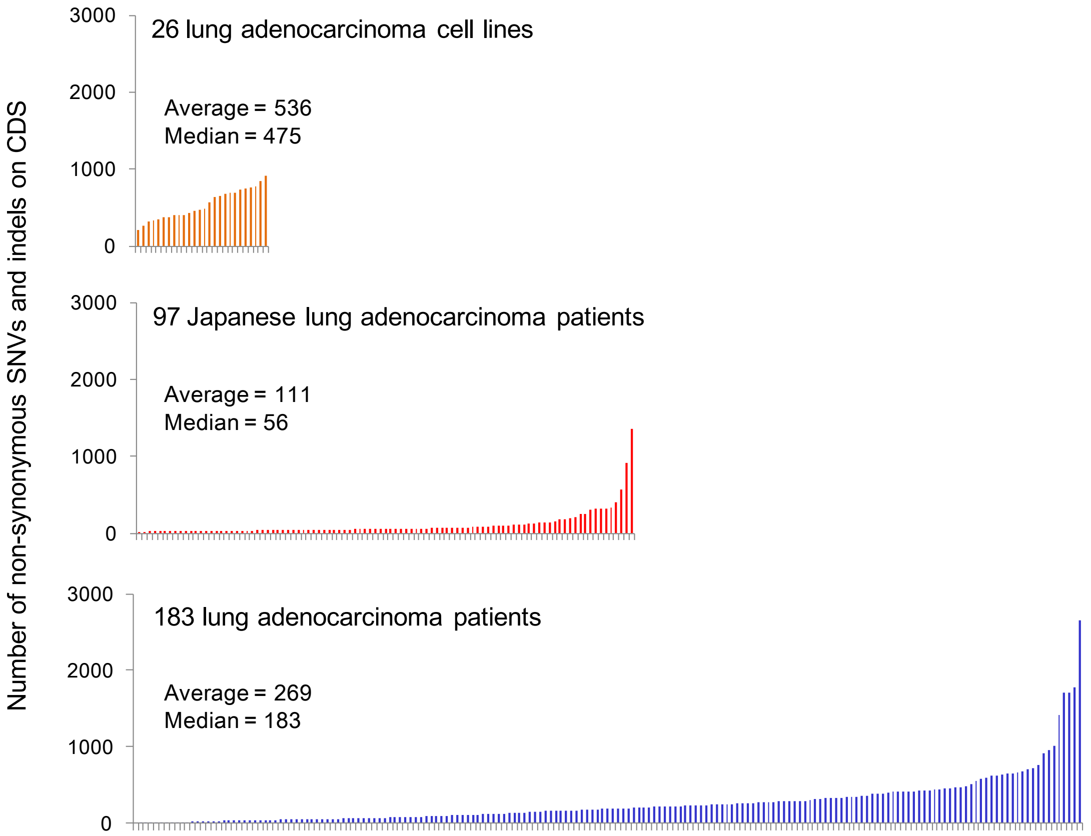

Supplementary Figure S8. The number of mutations compared with clinical sequencing data.

The numbers of non-synonymous SNVs and indels on CDS were visualized for each sample. The numbers of mutations of the 26 cell lines in this project, 97 Japanese patients from our recent study (10) and 183 patients from a previous study of other groups (11) are shown in upper, middle and lower panels, respectively. The x-axis is sorted by the number of mutations. The average and median numbers of mutations are shown in the inset.

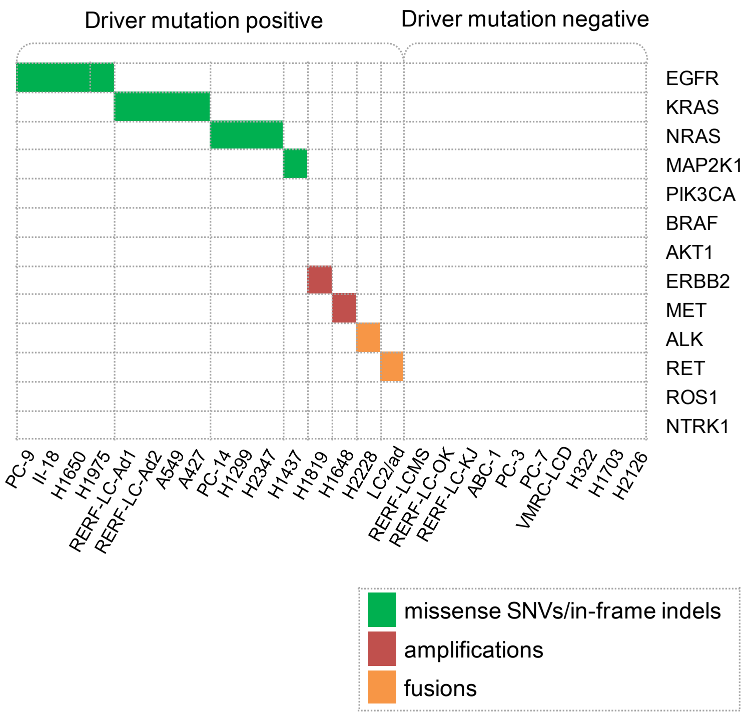

Supplementary Figure S9. The summary of driver mutations in the 26 cell lines.

Cell lines were divided into two groups; cell lines having previously reported driver mutations (16 cell lines) or not (10 cell lines). Driver mutations were listed as follows: L858R, G719A/C/S and E746_A750del (deletion in the 19th exon) in EGFR, G12/13 mutations in KRAS, Q61 mutations in NRAS, E542K, E545K/D/Q and H1047R/L in PIK3CA, V600E/K in BRAF, Q56P K57N, D67N in MAP2K1, RET fusions, ALK fusions, ROS1 fusions, NTRK1 fusions, EGFR amplifications, ERBB2 amplifications and MET amplifications.

<u>Summary of analysis</u>: We tried to identify previously unknown novel candidates of driver mutations using the data of 26 cell lines. For this purpose, we divided the 26 cell lines into two groups; cell lines having previously reported driver mutations (16 cell lines) or not (10 cell lines; **Supplementary Figure S9**). Assuming that unknown driver mutations are enriched in the latter group of the 10 cell lines (also note that most driver mutations are mutually exclusive within a given pathway or a functional category), we evaluated statistical enrichment of mutations within a particular functional domain of a gene. We detected 36 such mutations (in 18 genes) in the protein functional domains ($p < 0.05$) (**Supplementary Table S9A**; further details in procedure is described in the

legend). We particularly focused on mutations in kinase domains (IPR000719: Protein kinase, catalytic domain) and identified 18 mutations (17 genes; **Supplementary Table S9B**). We further examined and found that these mutation-harboring genes are transcribed by RNA-Seq (> 1 RPKM), thus, meet the criteria of the driver genes. Although further experimental validations should be needed, we believe these genes are good candidates for unknown driver mutations in these cell lines.

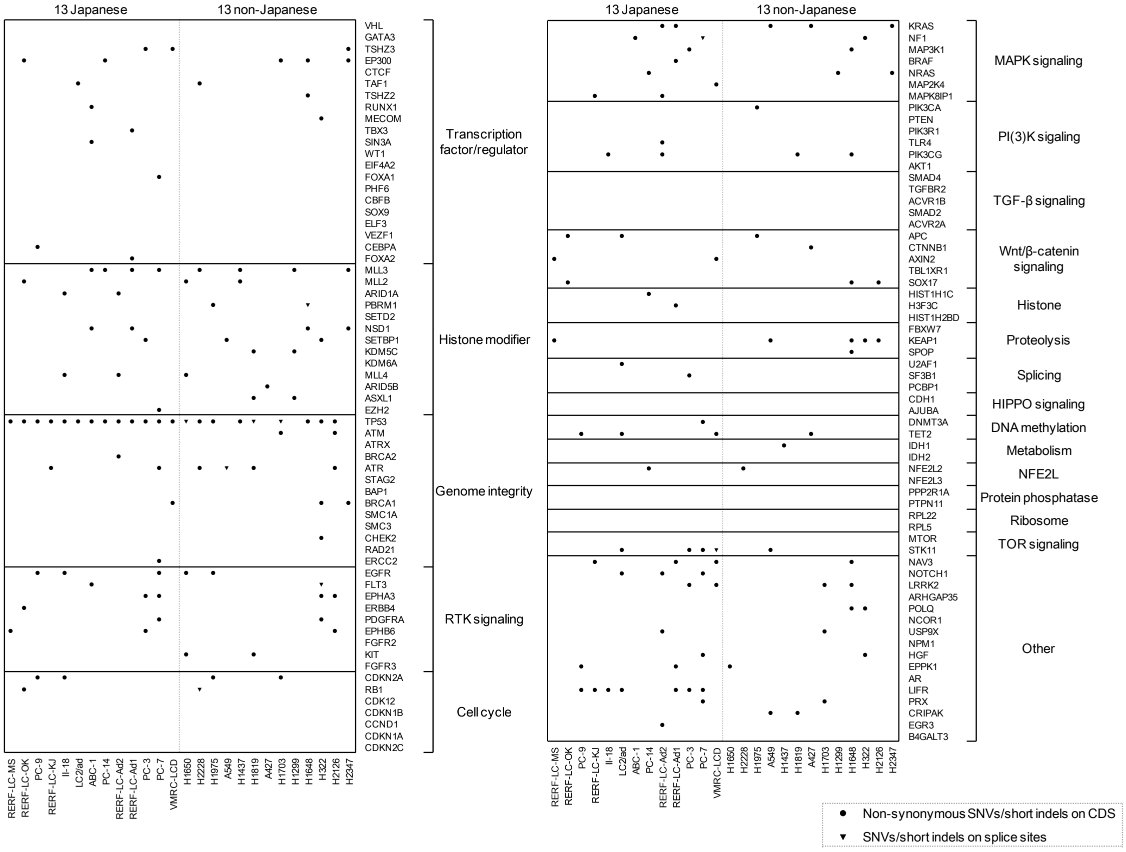

Supplementary Figure S10. Mutations in 125 cancer-related genes provided in TCGA. Non-synonymous SNVs and indels on CDS and splice site mutations were summarized for the 125 significantly mutated genes in 12 types of cancer types provided from TCGA paper (12). Two mutations in TP53 were added by manual inspection.

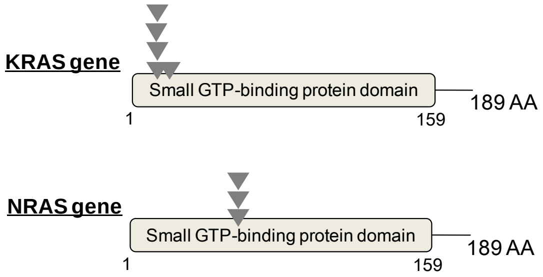

Supplementary Figure S11. Non-synonymous SNVs in the KRAS and NRAS genes. Non-synonymous SNVs in KRAS (upper panel) and NRAS (lower panel) are shown. In KRAS, four G12 mutations (G12A/D/V/S) and one L19F mutation were detected. In contrast, three Q61 mutations (two G61K and one Q61R mutations) were detected in NRAS.

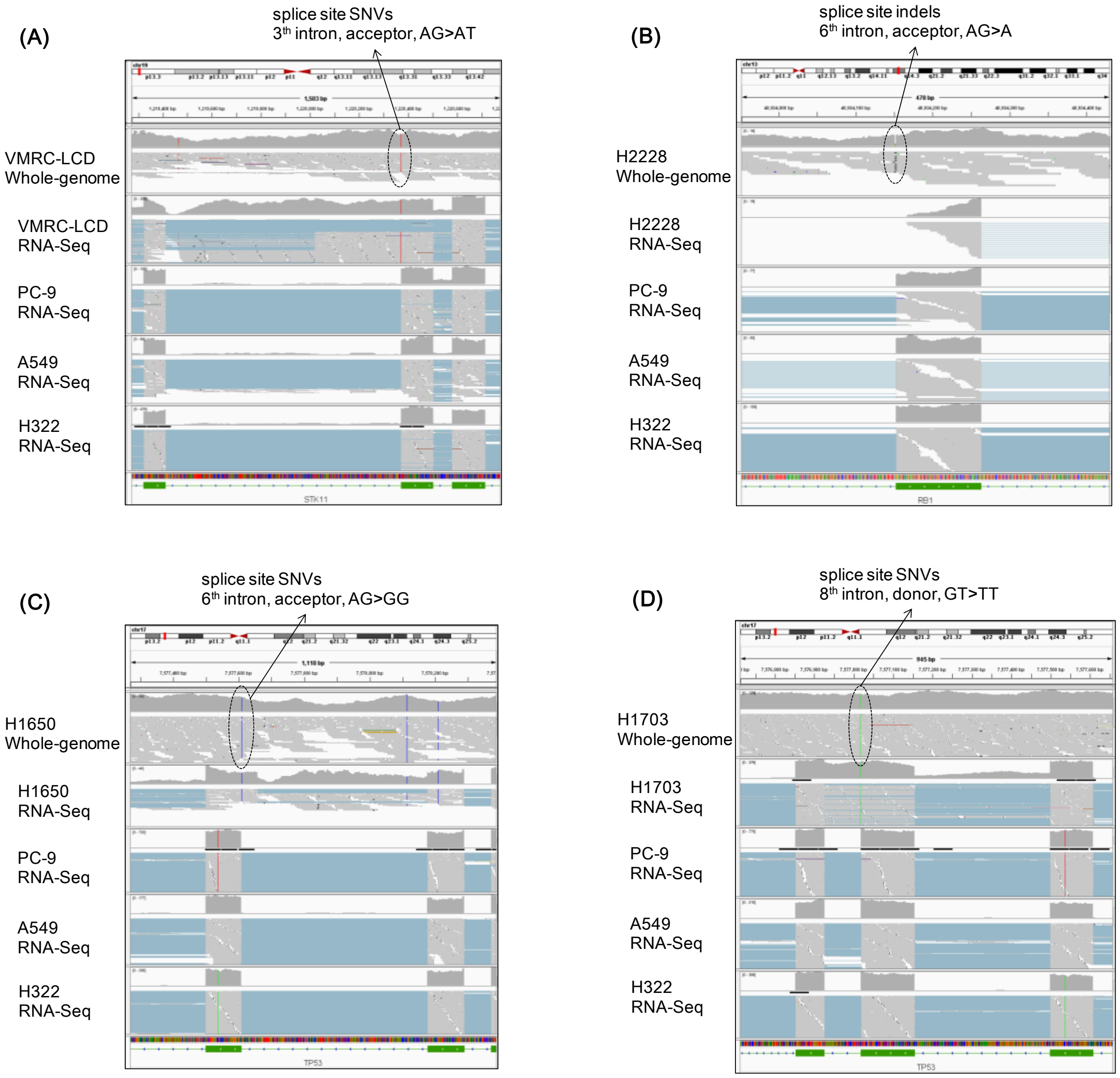

Supplementary Figure S12. Transcript consequences of the splice mutations in cancer-related genes.

Aberrant splicing events with splice site mutations in the cancer-related genes, especially tumor suppressor genes. For STK11 in VMRC-LCD cells (**A**), RB1 in H2228 cells (**B**), TP53 in H1650 cells (**C**) and TP53 in H1703 cells (**D**), IGV (13,14) visualizes splice site mutations in whole-genome sequences and the RNA-Seq compared with the RNA-Seq of PC-9, A549 and H322 cell lines as controls.

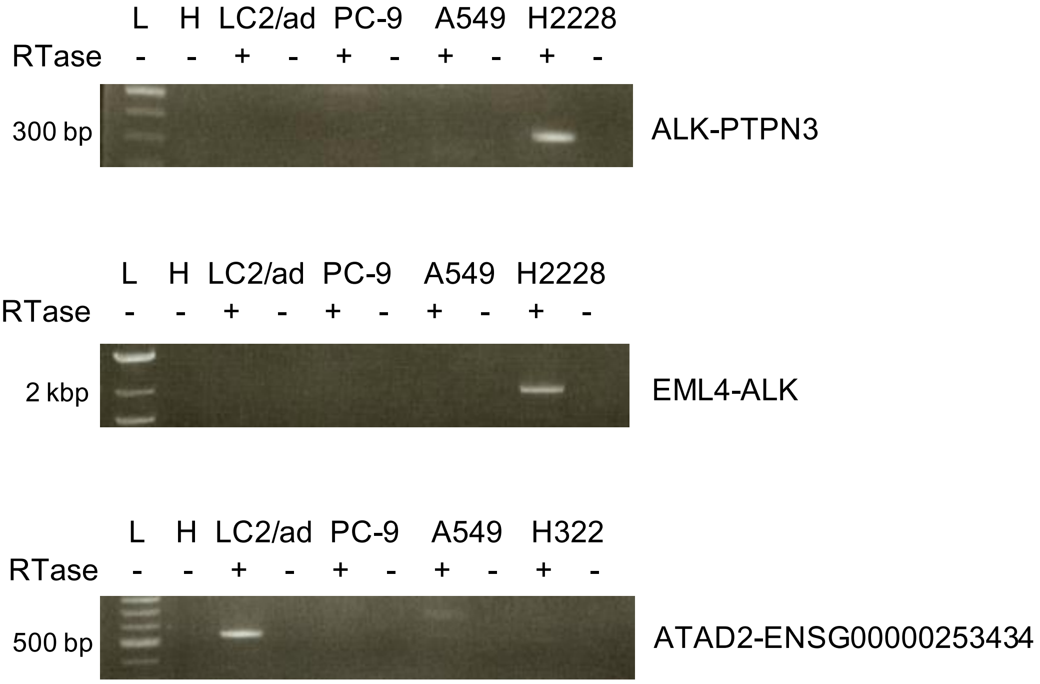

Supplementary Figure S13. RT-PCR validation for selected cases of fusion transcripts. Results of RT-PCR for the three fusion transcripts ALK-PTPN3 and EML4-ALK in the H2228 cell line and ATAD2-ENSG00000253434 in the LC2/ad cell line are shown. EML4-ALK fusion was detected by RT-PCR but not detected by the computational analysis (TopHat-fusion) (15) using RNA-seq data.

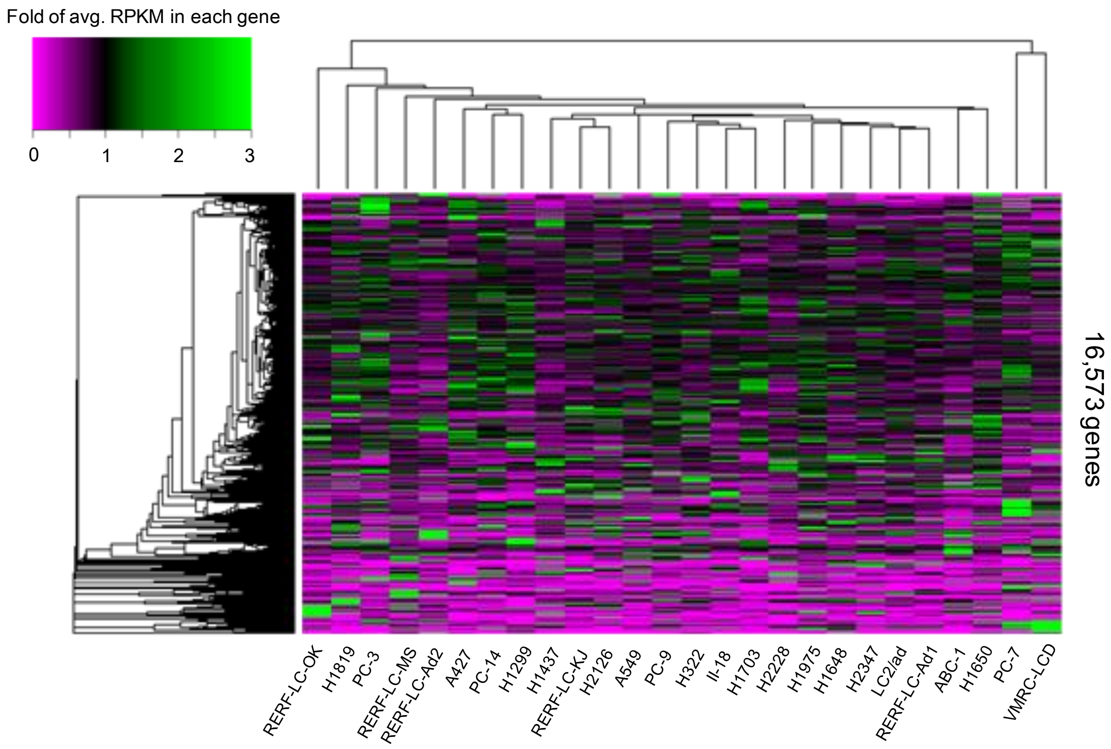

Supplementary Figure S14. Gene expression variations among the 26 cell lines.

Result of hierarchal clustering for 16,573 genes using RNA-Seq data is shown as a heat map.

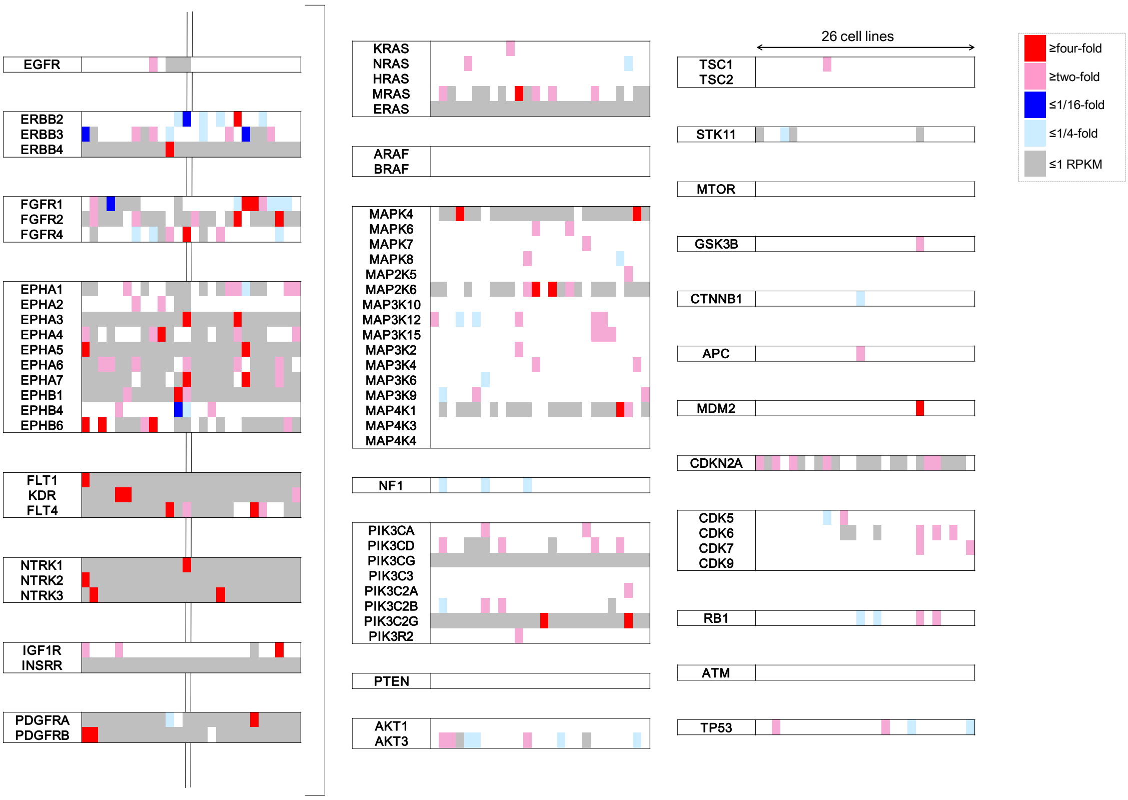

Supplementary Figure S15. Variable expression of the lung adenocarcinoma pathway. The variation of gene expression levels were mapped on the pathway of lung adenocarcinoma (16). The order of 26 cell lines in parallel was the same as in **Figure 1A**.

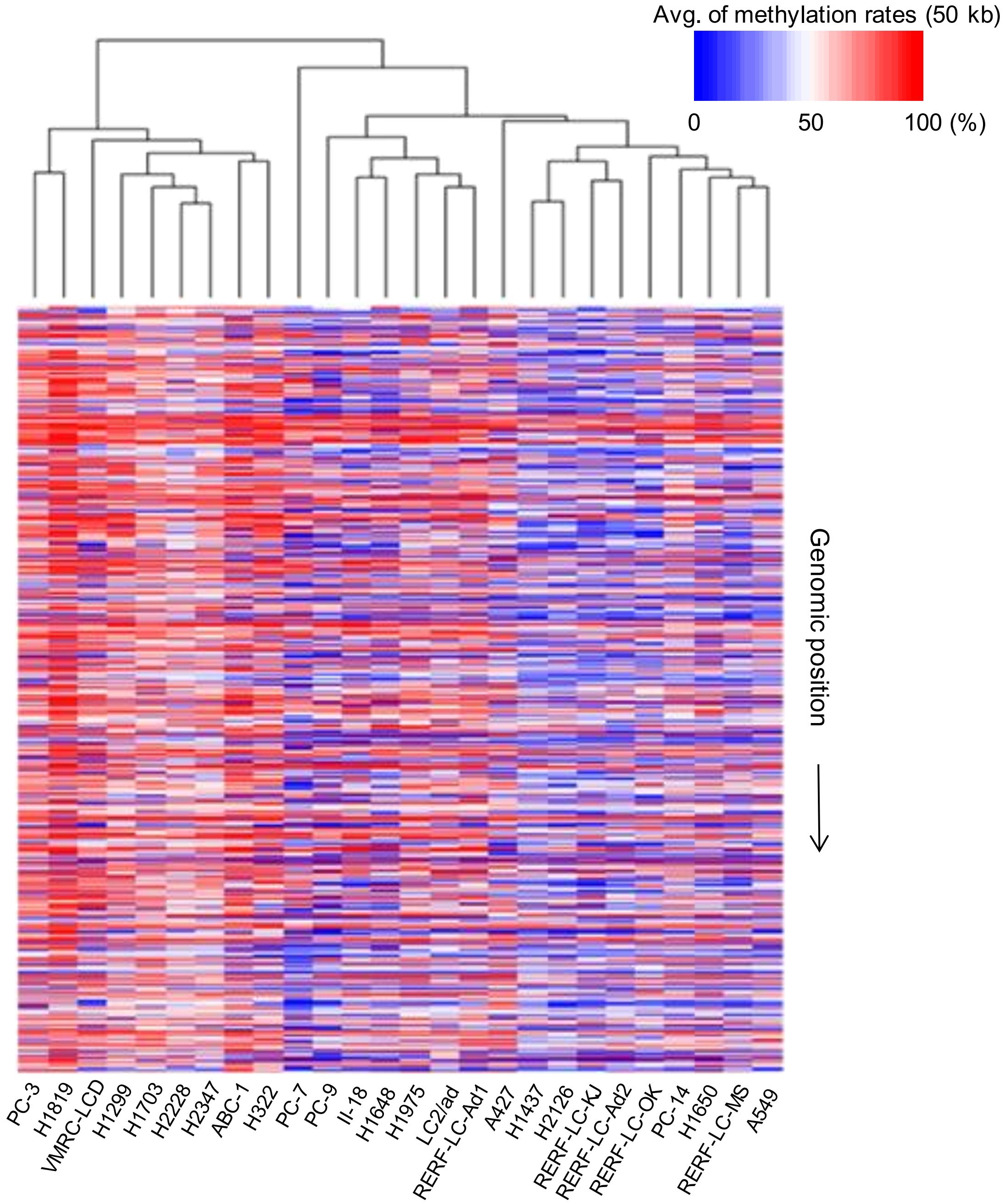

Supplementary Figure S16. Genome-wide DNA methylation patterns.

DNA methylation rates were calculated for each 50 kb of the human genome. The order of rows in the heat map was sorted by genomic positions that were supported by sufficient sequence tags. The cell lines were clustered by hierarchical clustering.

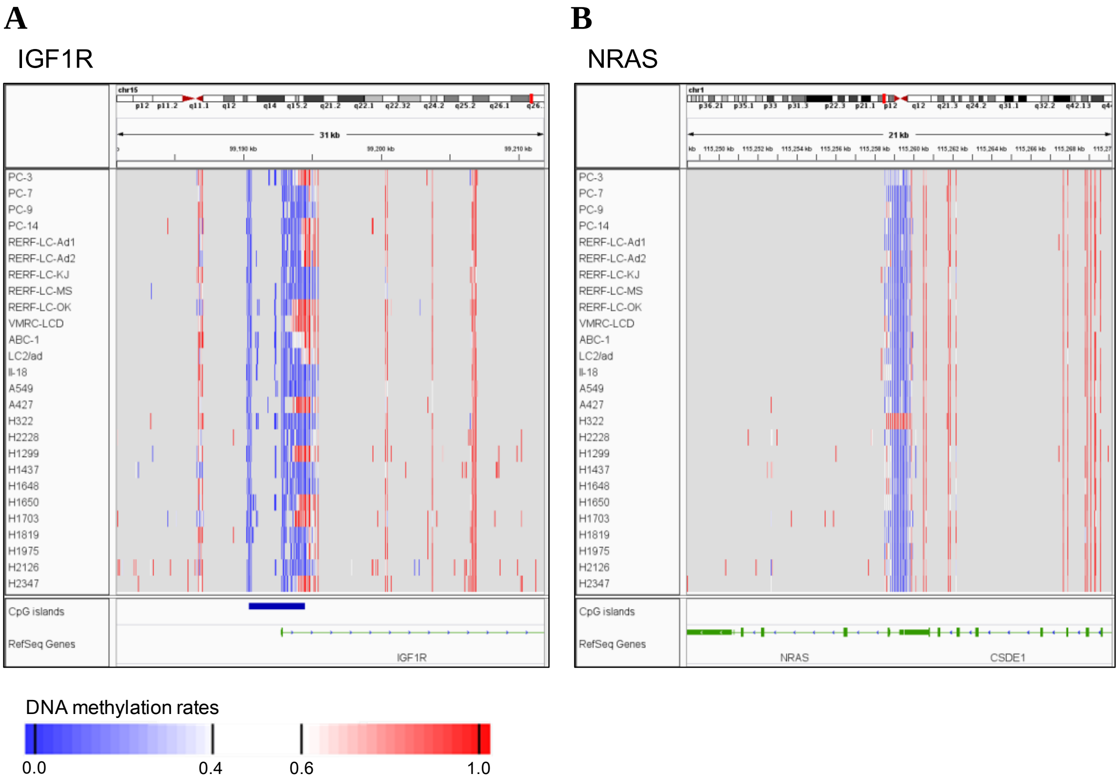

Supplementary Figure S17. DNA methylation on the IGF1R and NRAS promoters.
For the 26 cell lines, DNA methylation rates around the TSS of IGF1R (**A**) and NRAS (**B**) were visualized by IGV.

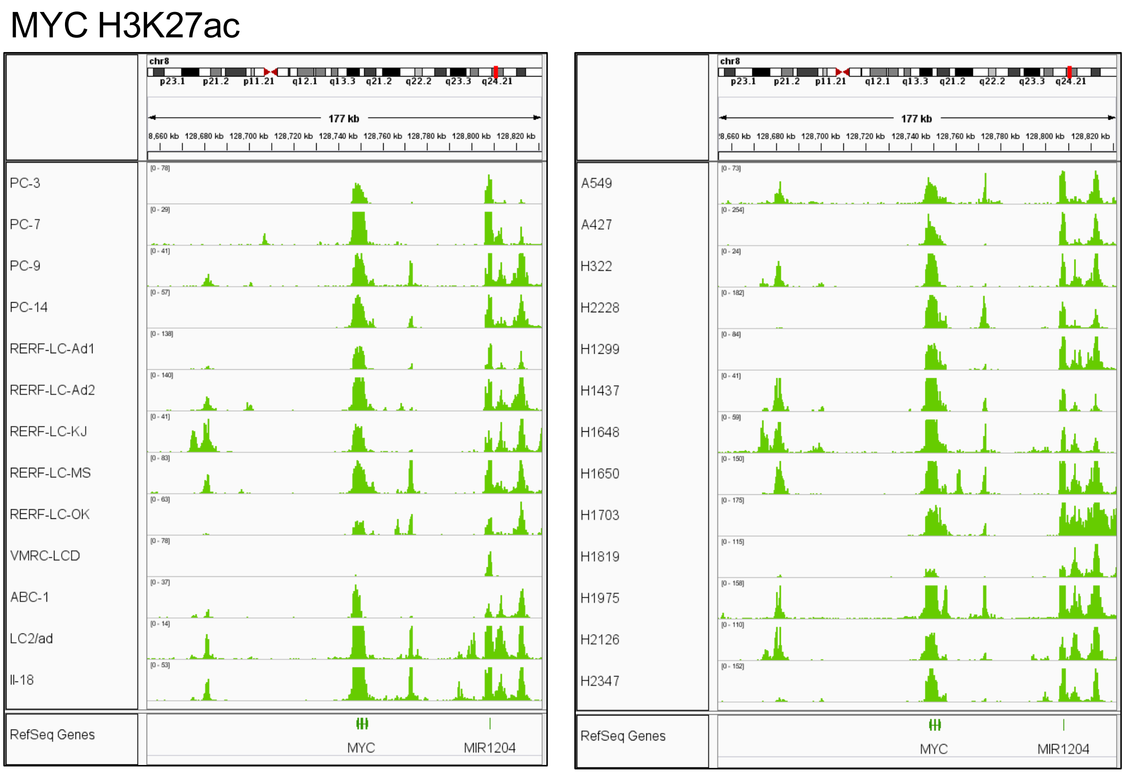

Supplementary Figure S18. An example of differentially utilized enhancers between cell lines.

H3K27ac marks around the MYC gene were visualized for each of the cell lines using IGV. Peak height represents the tag densities of ChIP-Seq signals.

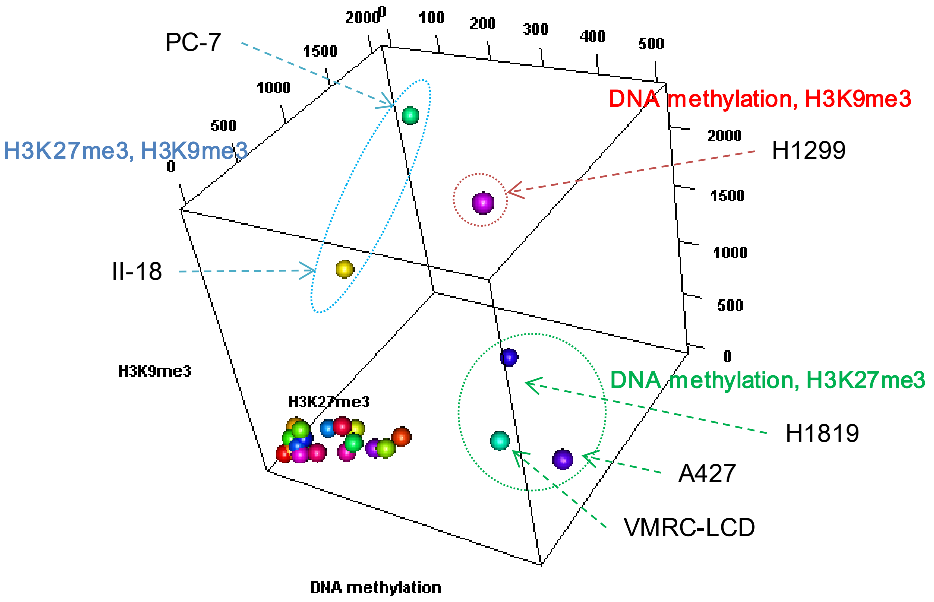

Supplementary Figure S19. Differential transcriptional repressive marks for each cell line.

For the 26 cell lines, the number of genes with differential higher (≥ 4-fold intensities of average) DNA methylation, H3K27me3 and H3K9me3 marks in the promoters were shown in the 3D plot. Each cell line was represented in each plot.

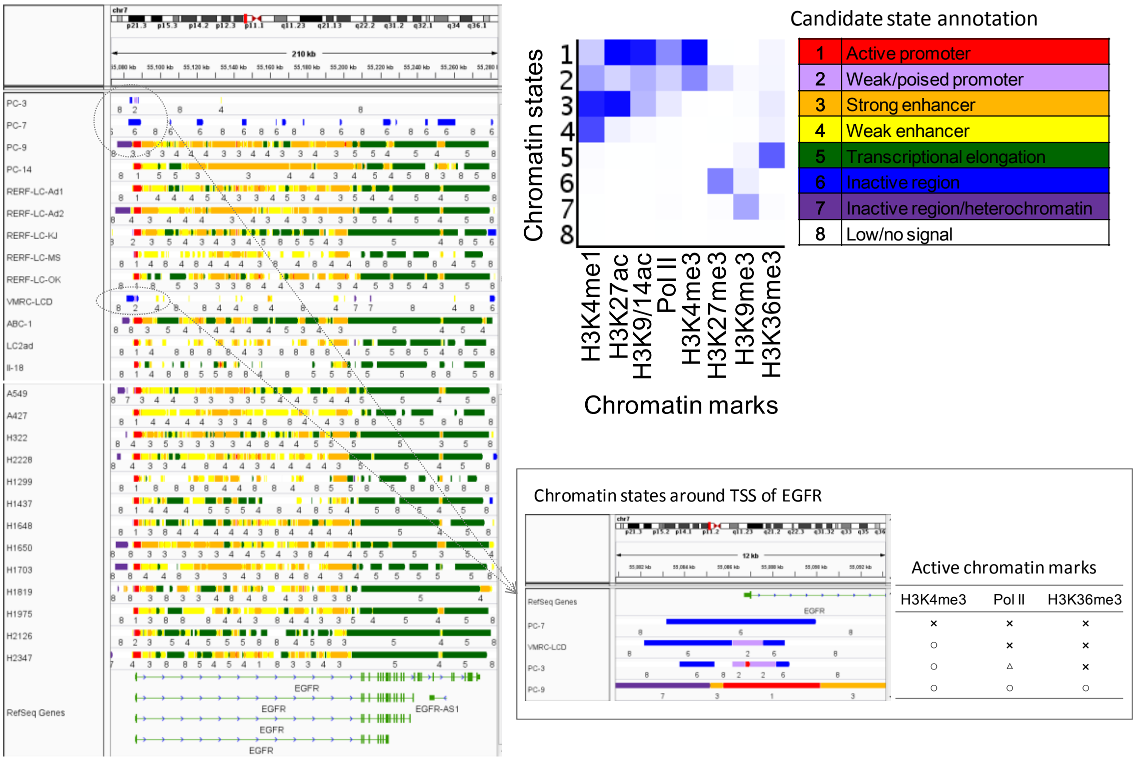

Supplementary Figure S20. Chromatin signature of EGFR visualized by ChromHMM. Using ChIP-Seq data of seven histone modifications and Pol II, eight chromatin states were discovered using ChromHMM (17,18). For example, the chromatin states of the EGFR gene were visualized by IGV.

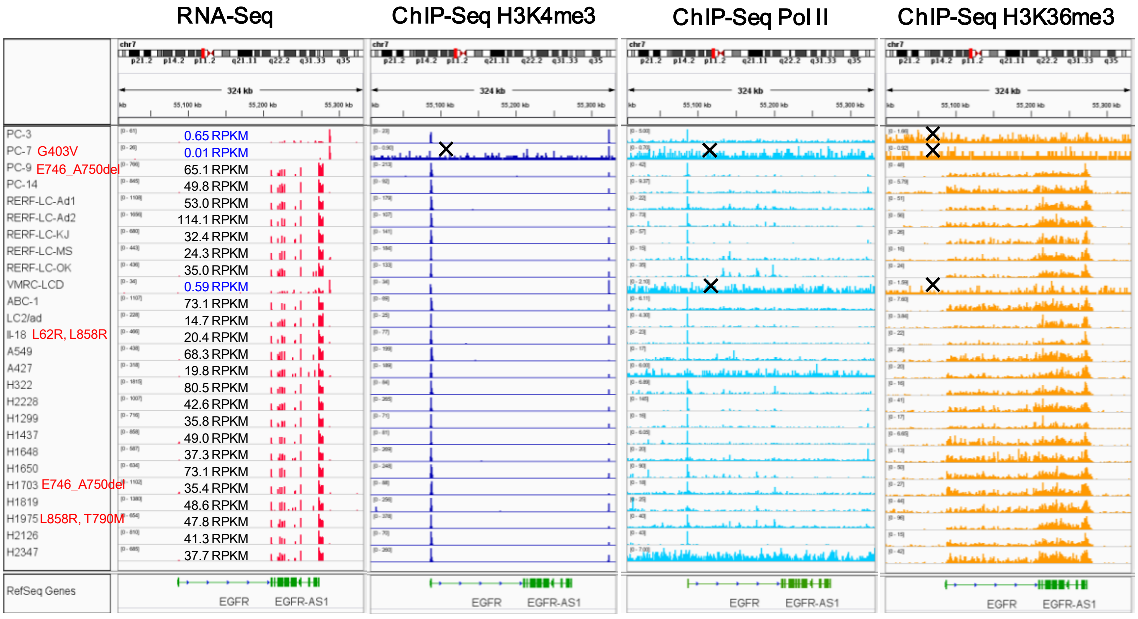

Supplementary Figure S21. Differential chromatin marks of EGFR.

For the EGFR gene, RNA-Seq and ChIP-seq for H3K4me3, Pol II and H3K36me3 were visualized on IGV (autoscale). X-marks mean no/low peaks of ChIP-Seq. SNVs and indels detected on CDS are shown in red letters. Three cell lines showed differential patterns of active chromatin marks (also see **Supplementary Figure S20**).

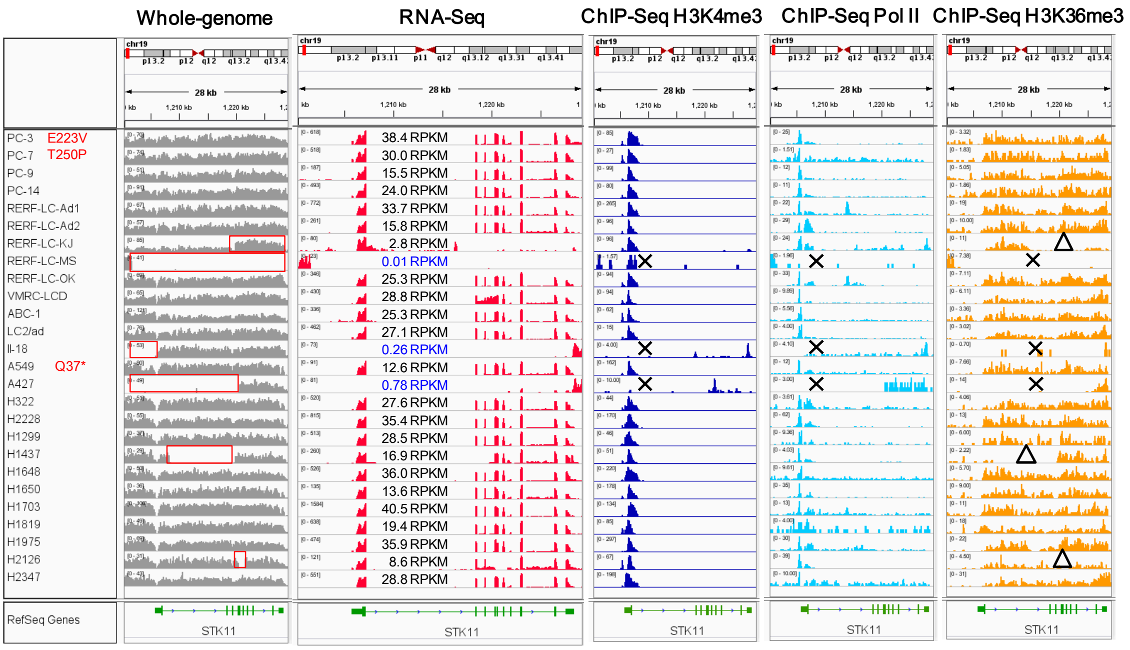

Supplementary Figure S22. Various patterns of genome, transcriptome and chromatin marks of STK11.

For the STK gene, whole-genome sequences, RNA-Seq and ChIP-seq for H3K4me3, Pol II and H3K36me3 were visualized on IGV (autoscale). X-marks mean no or low peaks of ChIP-Seq and triangles mean partly disrupted modifications. SNVs and indels detected on CDS (not benign, PolyPhen-2) are shown in red letters. Several cell lines showed various patterns of genomic aberrations, which caused transcriptomic and epigenomic aberrations.

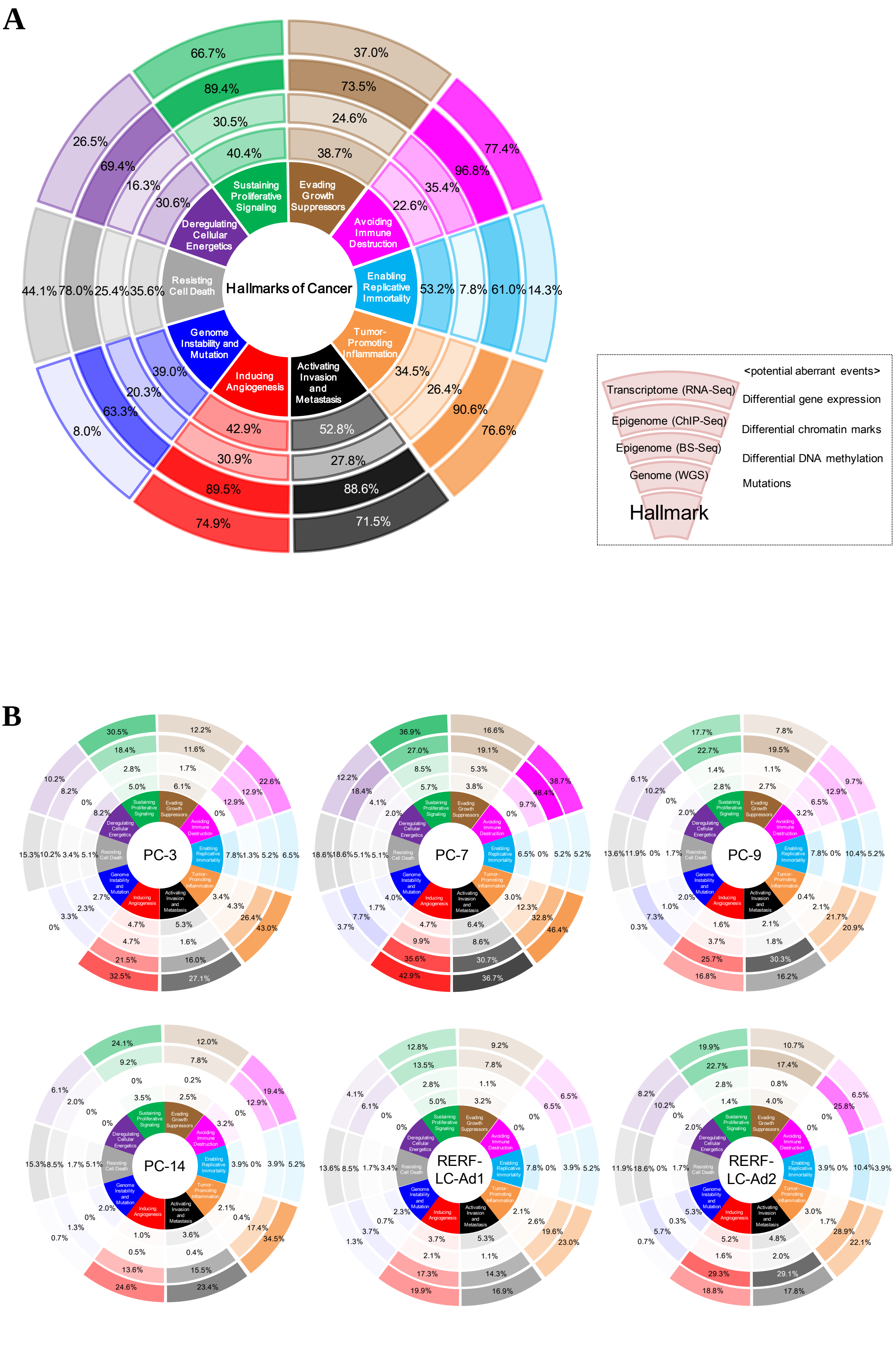
A
Hallmarks of Cancer
Sustaining Proliferative Signaling
66.7%
89.4%
30.5%
40.4%
Evading Growth Suppressors
37.0%
73.5%
24.6%
38.7%
Avoiding Immune Destruction
77.4%
96.8%
35.4%
22.6%
Enabling Replicative Immortality
53.2%
7.8%
61.0%
14.3%
Tumor-Promoting Inflammation
34.5%
26.4%
90.6%
76.6%
Activating Invasion and Metastasis
52.8%
27.8%
88.6%
71.5%
Inducing Angiogenesis
42.9%
30.9%
89.5%
74.9%
Genome Instability and Mutation
39.0%
20.3%
63.3%
8.0%
Resisting Cell Death
44.1%
78.0%
25.4%
35.6%
Deregulating Cellular Energetics
26.5%
69.4%
16.3%
30.6%
<potential aberrant events>
Transcriptome (RNA-Seq)
Differential gene expression
Epigenome (ChIP-Seq)
Differential chromatin marks
Epigenome (BS-Seq)
Differential DNA methylation
Genome (WGS)
Mutations
Hallmark
B
PC-3
PC-7
PC-9
PC-14
RERF-LC-Ad1
RERF-LC-Ad2

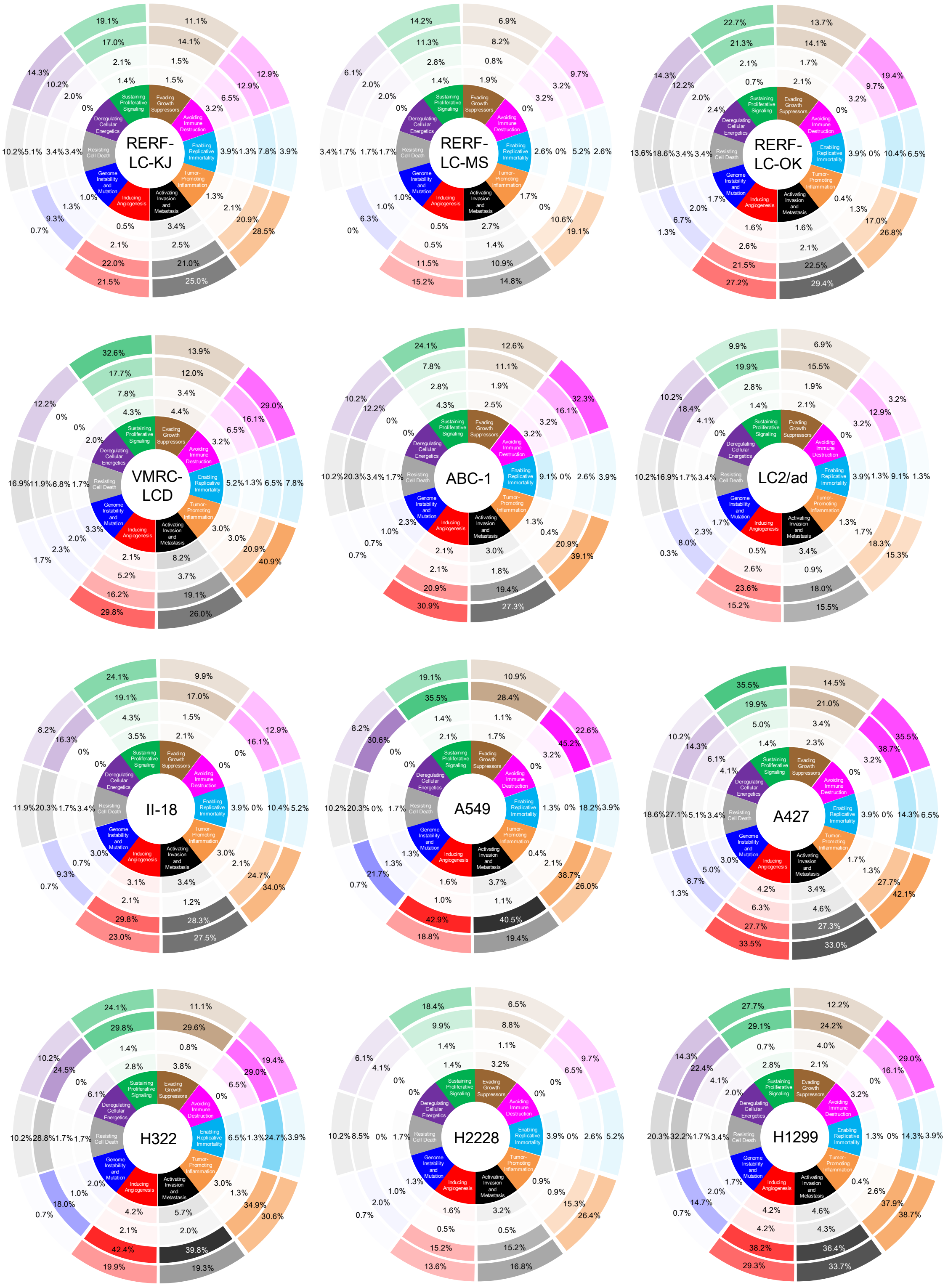
RERF-LC-KJ
RERF-LC-MS
RERF-LC-OK
VMRC-LCD
ABC-1
LC2/ad
II-18
A549
A427
H322
H2228
H1299
Sustaining Proliferative Signaling
Evading Growth Suppressors
Avoiding Immune Destruction
Enabling Replicative Immortality
Tumor-Promoting Inflammation
Activating Invasion and Metastasis
Inducing Angiogenesis
Genome Instability and Mutation
Resisting Cell Death
Deregulating Cellular Energetics

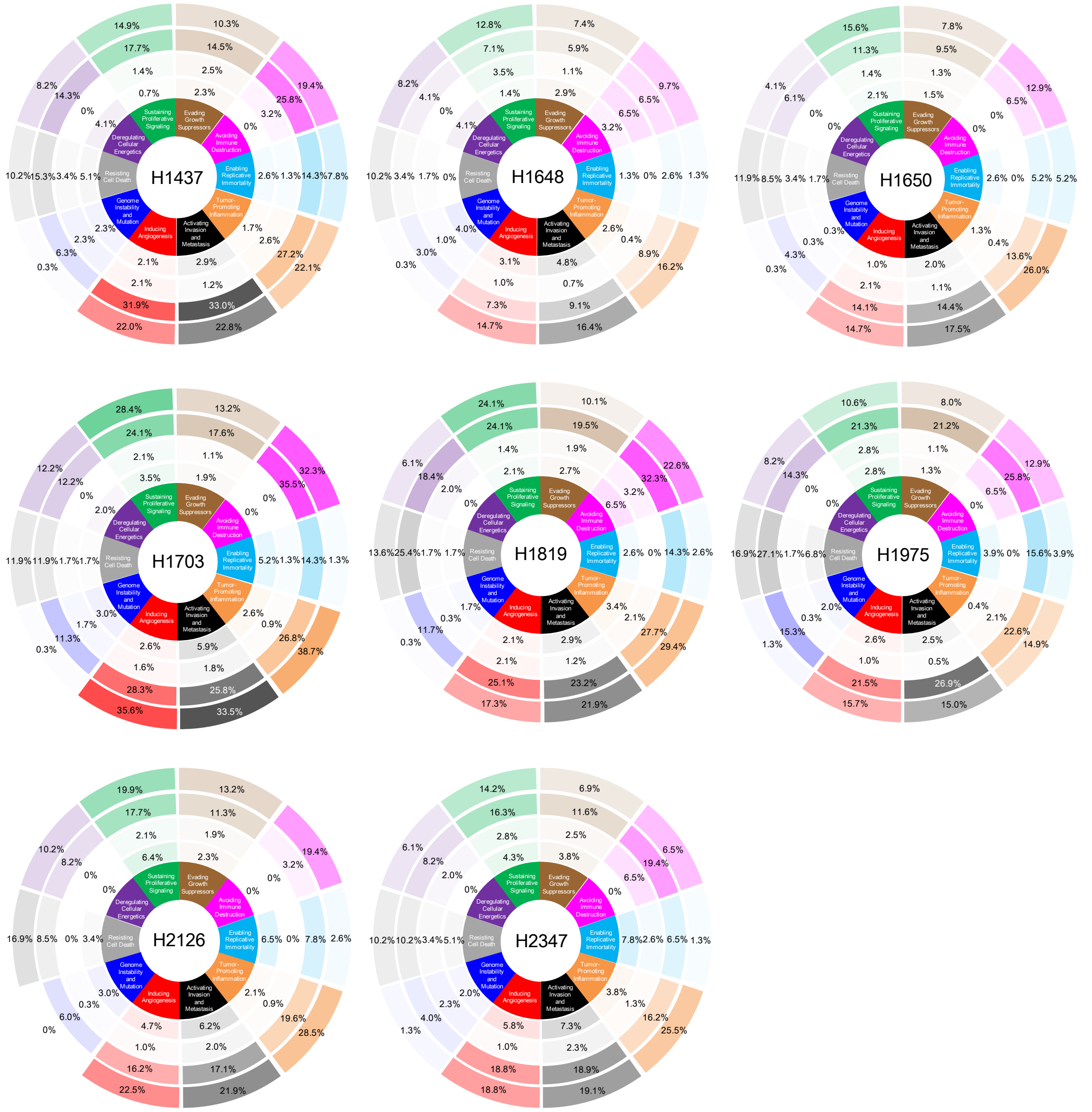

Supplementary Figure S23. Differential aberrant events in the "hallmarks of cancer". Percentages of genes with potential aberrant events (shown in the inset) in genome, epigenome and transcriptome were shown for each hallmark of cancer (19) for all the 26 cell lines (**A**) and each cell line (**B**). We used transcribed genes (>1 RPKM) in at least one cell line.

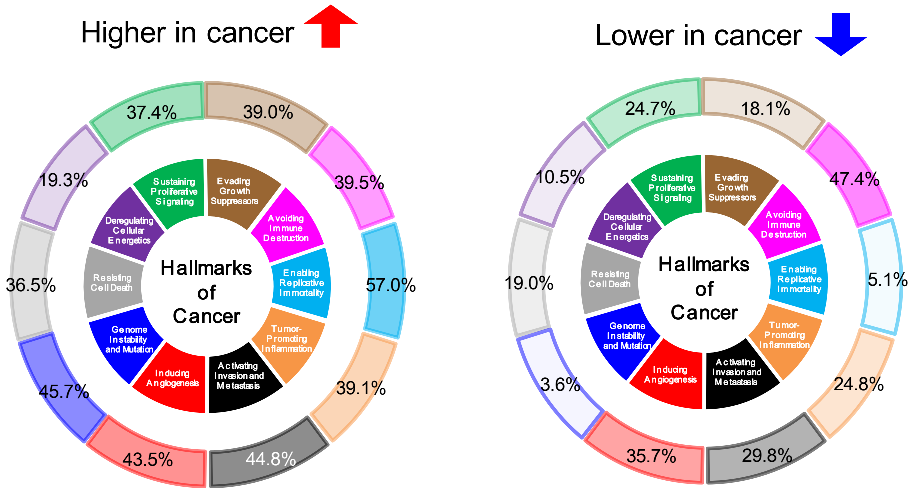

Supplementary Figure S24. Differential gene expression of the cancer cell lines comparing SAEC in "hallmarks of cancer".

Percentages of genes with higher (left) or lower (right) expression levels than those of SAEC were represented for each hallmark.

SUPPLEMENTARY TABLES

Supplementary Table S1. List of the cancer cell lines used in this study.

Cell line	Ethnicity	Distributor*	No.
PC-3	Japanese	JCRB	JCRB0077
PC-7	Japanese	IBL	Upon request to us.
PC-9	Japanese	RIKEN BRC	RCB4455
PC-14	Japanese	IBL	Upon request to us.
RERF-LC-Ad1	Japanese	JCRB	JCRB1020
RERF-LC-Ad2	Japanese	JCRB	JCRB1021
RERF-LC-KJ	Japanese	RIKEN BRC	RCB1313
RERF-LC-MS	Japanese	JCRB	JCRB0081
RERF-LC-OK	Japanese	JCRB	JCRB0811
VMRC-LCD	Japanese	JCRB	JCRB0814
ABC-1	Japanese	JCRB	JCRB0815
LC2/ad	Japanese	RIKEN BRC	RCB0440
II-18	Japanese	RIKEN BRC	RCB2093
A549	Caucasian†	ATCC	CCL-185
A427	Caucasian†	ATCC	HTB-53
H322	Caucasian†	ATCC	CRL-5806
H2228	Unknown†	ATCC	CRL-5935
H1299	Caucasian†	ATCC	CRL-5803
H1437	Caucasian†	ATCC	CRL-5872
H1648	Black†	ATCC	CRL-5882
H1650	Caucasian†	ATCC	CRL-5883
H1703	Caucasian†	ATCC	CRL-5889
H1819	Caucasian†	ATCC	CRL-5897
H1975	Unknown†	ATCC	CRL-5908
H2126	Caucasian†	ATCC	CCL-256
H2347	Caucasian†	ATCC	CRL-5942

*IBL: Immuno-Biological Laboratories; JCRB: Japanese Collection of Research Bioresources; RIKEN BRC: RIKEN Bio Resource Center; ATCC: American Type Culture Collection.

†Refer to the ATCC webpage (http://www.atcc.org/).

Supplementary Table S6. Remaining germline variants in this datasets.

	H1437 (BL1437*)	H2126 (BL2126*)	H2347 (BL2347*)	Average of three cell lines
Number of mutations				
Somatic candidates†	123,585	203,979	227,518	185,027
Somatic‡	77,917	160,984	171,432	136,778
Remaining germline	45,668	42,995	56,086	46,250
(%)	(37.0%)	(21.1%)	(24.7%)	(27.6%)
Number of non-synonymous SNVs and indels on CDS				
Somatic candidates†	397	770	917	694
Somatic‡	273	651	768	564
Remaining germline	124	119	149	130
(%)	(31.2%)	(15.5%)	(16.2%)	(21.0%)

*Normal counterparts derived from B lymphoblasts and provided from ATCC.

†Detected using the pipeline as shown in **Supplementary Figure S1**.

‡After removing germline mutations using whole-genome sequencing data from the normal counterpart.

Supplementary Table S7. Copy number information in the 26 cell lines.

Cell line	Copy number gains*		Copy number losses*	
	Total	Gene region	Total	Gene region
PC-3	316	133	148	95
PC-7	366	202	133	79
PC-9	265	107	187	127
PC-14	326	124	198	120
RERF-LC-Ad1	332	102	175	104
RERF-LC-Ad2	326	170	81	52
RERF-LC-KJ	294	126	198	129
RERF-LC-MS	343	102	140	76
RERF-LC-OK	345	253	117	82
VMRC-LCD	336	158	188	133
LC2/ad	310	142	146	94
ABC-1	274	92	174	100
II-18	364	284	114	81
A549	168	79	140	90
A427	311	119	210	133
H322	344	163	159	103
H2228	325	86	243	153
H1299	303	109	204	118
H1437	346	191	149	105
H1648	290	126	139	86
H1650	315	113	237	132
H1703	274	220	52	33
H1819	435	199	217	114
H1975	348	124	139	74
H2126	325	132	199	115
H2347	312	85	201	117

*Copy number gains: normalized copy number ≥ 4; copy number losses: normalized copy number < 2.

Supplementary Table S8. Genomic rearrangements computationally detected in this datasets.

Cell line	Number of rearrangements
PC-3	4
PC-7	11
PC-9	1
PC-14	7
RERF-LC-Ad1	1
RERF-LC-Ad2	13
RERF-LC-KJ	2
RERF-LC-MS	3
RERF-LC-OK	91
VMRC-LCD	5
ABC-1	6
LC2/ad	4
II-18	130
A549	74
A427	9
H322	9
H2228	11
H1299	6
H1437	8
H1648	4
H1650	3
H1703	128
H1819	9
H1975	5
H2126	4
H2347	4

Supplementary Table S10. Summary of the RNA-Seq data.

Cell line	Used sequences (Read1)	Num of genes	
		> 1 RPKM	> 5 RPKM
PC-3	49,914,547	12,205	9,240
PC-7	50,925,975	12,129	9,009
PC-9	34,167,521	12,817	9,532
PC-14	53,977,381	12,169	9,037
RERF-LC-Ad1	56,406,046	12,298	9,206
RERF-LC-Ad2	45,580,359	12,392	8,804
RERF-LC-KJ	60,803,665	12,054	8,938
RERF-LC-MS	52,715,099	13,045	9,090
RERF-LC-OK	33,086,988	12,309	8,954
VMRC-LCD	45,944,953	12,502	8,711
ABC-1	37,993,504	11,715	8,384
LC2/ad	43,665,988	12,366	9,206
II-18	63,869,445	11,955	9,038
A549	20,440,396	12,155	8,998
A427	41,895,881	11,866	9,011
H322	54,487,583	12,457	9,351
H2228	56,465,940	12,409	9,106
H1299	51,120,991	11,735	8,958
H1437	49,890,034	12,275	8,921
H1648	38,908,100	12,604	9,317
H1650	26,635,691	12,716	9,595
H1703	87,705,180	11,736	8,695
H1819	75,262,673	12,494	9,185
H1975	36,195,247	12,715	9,634
H2126	46,862,796	12,143	9,016
H2347	50,325,156	12,278	9,030
SAEC	180,054,144	12,126	8,809

Supplementary Table S11. Summary of bisulfite sequencing data.

Cell line	Mapped sequences	Avg. of depths	Conversion rate (x5)*	CpG sites (> x5)
PC-3	157,902,653	161.4	0.994	3,673,159
PC-7	109,919,011	110.9	0.994	3,418,929
PC-9	87,012,056	89.6	0.994	3,231,320
PC-14	204,216,479	210.3	0.994	4,064,068
RERF-LC-Ad1	87,043,746	89.1	0.992	3,264,395
RERF-LC-Ad2	78,300,691	83.0	0.994	3,448,211
RERF-LC-KJ	72,844,738	74.9	0.993	3,068,971
RERF-LC-MS	102,938,936	109.0	0.994	3,598,662
RERF-LC-OK	161,552,507	165.0	0.993	3,758,532
VMRC-LCD	84,681,570	89.5	0.992	3,136,774
LC2/ad	112,097,386	116.0	0.988	3,548,548
ABC-1	93,158,547	93.1	0.993	3,493,903
II-18	99,682,438	165.0	0.993	3,327,001
A549	87,966,180	91.0	0.991	3,324,364
A427	53,499,542	54.3	0.992	2,614,641
H322	153,896,186	165.8	0.989	4,161,775
H2228	122,705,759	81.6	0.993	4,815,543
H1299	118,923,875	82.2	0.994	4,533,930
H1437	98,311,209	63.1	0.993	4,382,225
H1648	102,033,841	104.4	0.989	3,357,747
H1650	105,694,196	109.4	0.994	3,460,378
H1703	127,897,486	81.6	0.994	5,513,896
H1819	220,008,485	223.4	0.986	4,085,231
H1975	79,688,628	81.7	0.993	3,274,116
H2126	124,651,437	80.2	0.993	4,991,289
H2347	115,973,241	76.1	0.993	4,661,415

*Conversion rate: (TA+TT+TC) / (CA+CT+CC+TA+TT+TC).

Supplementary Table S12. Summary of ChIP-Seq.

A. 26 cancer cell lines

ChIP	Average of mapped sequences	Average of number of peaks (MACS2)	
		Narrow peaks	Broad peaks
H3K4me3	26,140,455	21,209	16,208
H3K9/14ac	19,596,187	34,374	23,753
Pol II	26,056,772	15,715	13,997
H3K36me3	24,264,604	107,708	47,710
H3K4me1	25,900,257	108,882	75,854
H3K27ac	25,690,276	61,061	38,297
H3K27me3	21,584,812	53,587	42,163
H3K9me3	21,155,573	39,559	51,760
WCE	19,100,553	***	***

B. SAEC

ChIP	Mapped sequences	Average of number of peaks (MACS2)	
		Narrow peaks	Broad peaks
H3K4me3	43,579,277	15,626	14,093
H3K9/14ac	21,603,337	50,674	45,159
Pol II	21,986,637	16,703	15,234
H3K36me3	56,493,935	321,485	107,145
H3K4me1	52,851,492	226,330	154,297
H3K27ac	45,848,952	170,013	88,659
H3K27me3	29,626,299	88,943	83,095
H3K9me3	40,496,823	316,142	148,544
WCE	45,429,763	***	***

REFERENCES

1. Li, H. and Durbin, R. (2009) Fast and accurate short read alignment with Burrows-Wheeler transform. *Bioinformatics*, **25**, 1754-1760.
2. Li, H., Handsaker, B., Wysoker, A., Fennell, T., Ruan, J., Homer, N., Marth, G., Abecasis, G. and Durbin, R. (2009) The Sequence Alignment/Map format and SAMtools. *Bioinformatics*, **25**, 2078-2079.
3. McKenna, A., Hanna, M., Banks, E., Sivachenko, A., Cibulskis, K., Kernytsky, A., Garimella, K., Altshuler, D., Gabriel, S., Daly, M. *et al.* (2010) The Genome Analysis Toolkit: a MapReduce framework for analyzing next-generation DNA sequencing data. *Genome Res*, **20**, 1297-1303.
4. DePristo, M.A., Banks, E., Poplin, R., Garimella, K.V., Maguire, J.R., Hartl, C., Philippakis, A.A., del Angel, G., Rivas, M.A., Hanna, M. *et al.* (2011) A framework for variation discovery and genotyping using next-generation DNA sequencing data. *Nat Genet*, **43**, 491-498.
5. Forbes, S.A., Bhamra, G., Bamford, S., Dawson, E., Kok, C., Clements, J., Menzies, A., Teague, J.W., Futreal, P.A. and Stratton, M.R. (2008) The Catalogue of Somatic Mutations in Cancer (COSMIC). *Curr Protoc Hum Genet*, **Chapter 10**, Unit 10 11.
6. Forbes, S.A., Bindal, N., Bamford, S., Cole, C., Kok, C.Y., Beare, D., Jia, M., Shepherd, R., Leung, K., Menzies, A. *et al.* (2011) COSMIC: mining complete cancer genomes in the Catalogue of Somatic Mutations in Cancer. *Nucleic Acids Res*, **39**, D945-950.
7. Sherry, S.T., Ward, M.H., Kholodov, M., Baker, J., Phan, L., Smigielski, E.M. and Sirotkin, K. (2001) dbSNP: the NCBI database of genetic variation. *Nucleic Acids Res*, **29**, 308-311.
8. NHLBI GO Exome Sequencing Project (ESP). Exome Variant Server. Seattle, WA (URL: http://evs.gs.washington.edu/EVS/) [8th (Oct, 2013) accessed].
9. The 1000 Genomes Project Consortium. (2012) An integrated map of genetic variation from 1,092 human genomes. *Nature*, **491**, 56-65.
10. Suzuki, A., Mimaki, S., Yamane, Y., Kawase, A., Matsushima, K., Suzuki, M., Goto, K., Sugano, S., Esumi, H., Suzuki, Y. *et al.* (2013) Identification and characterization of cancer mutations in Japanese lung adenocarcinoma without sequencing of normal tissue counterparts. *PLoS One*, **8**, e73484.
11. Imielinski, M., Berger, A.H., Hammerman, P.S., Hernandez, B., Pugh, T.J., Hodis, E., Cho, J., Suh, J., Capelletti, M., Sivachenko, A. *et al.* (2012) Mapping the hallmarks of lung adenocarcinoma with massively parallel sequencing. *Cell*, **150**, 1107-1120.
12. Kandoth, C., McLellan, M.D., Vandin, F., Ye, K., Niu, B., Lu, C., Xie, M., Zhang, Q.,

McMichael, J.F., Wyczalkowski, M.A. *et al.* (2013) Mutational landscape and significance across 12 major cancer types. *Nature*, **502**, 333-339.

13. Robinson, J.T., Thorvaldsdóttir, H., Winckler, W., Guttman, M., Lander, E.S., Getz, G. and Mesirov, J.P. (2011) Integrative genomics viewer. *Nat Biotechnol*, **29**, 24-26.
14. Thorvaldsdóttir, H., Robinson, J.T. and Mesirov, J.P. (2013) Integrative Genomics Viewer (IGV): high-performance genomics data visualization and exploration. *Brief Bioinform*, **14**, 178-192.
15. Kim, D. and Salzberg, S.L. (2011) TopHat-Fusion: an algorithm for discovery of novel fusion transcripts. *Genome Biol*, **12**, R72.
16. Ding, L., Getz, G., Wheeler, D.A., Mardis, E.R., McLellan, M.D., Cibulskis, K., Sougnez, C., Greulich, H., Muzny, D.M., Morgan, M.B. *et al.* (2008) Somatic mutations affect key pathways in lung adenocarcinoma. *Nature*, **455**, 1069-1075.
17. Ernst, J. and Kellis, M. (2012) ChromHMM: automating chromatin-state discovery and characterization. *Nat Methods*, **9**, 215-216.
18. Ernst, J., Kheradpour, P., Mikkelsen, T.S., Shoresh, N., Ward, L.D., Epstein, C.B., Zhang, X., Wang, L., Issner, R., Coyne, M. *et al.* (2011) Mapping and analysis of chromatin state dynamics in nine human cell types. *Nature*, **473**, 43-49.
19. Hanahan, D. and Weinberg, R.A. (2011) Hallmarks of cancer: the next generation. *Cell*, **144**, 646-674.
